# Supplementary material for: Highly efficient dual photoredox/copper catalyzed atom transfer radical polymerization achieved through mechanism-driven photocatalyst design
Source: Nat Commun. 2024 Jun 17;15:5160. doi: 10.1038/s41467-024-49509-1 (PMC11183263; doi:10.1038/s41467-024-49509-1)
Supplement: Supplementary file 1 — Supplementary Information [file 41467_2024_49509_MOESM1_ESM.pdf]

Supplementary Information

Highly efficient dual photoredox/copper catalyzed atom transfer radical polymerization achieved through mechanism-driven photocatalyst design

Woojin Jeon[1], Yonghwan Kwon[1,*], and Min Sang Kwon[1,*]

[1]Department of Materials Science and Engineering and Research Institute of Advanced Materials, Seoul National University, Seoul 08826, Republic of Korea

e-mail: minsang@snu.ac.kr (M. S. K.); yhkwon1995@snu.ac.kr (Y. K.)

Contents

Supplementary Note 1. General information 3

1.1. Chemicals 3

1.2. General experimental procedures 3

1.3 Graphical supplementary information for general procedure 5

1.4 Instrumentation 6

1.5 Computational methods 7

1.6 Synthesis of PCs 8

Supplementary Note 2. Characterization of PCs 10

2.1. Previously reported ATRP with photoredox/copper dual catalysis 10

2.2. Properties of PCs 13

2.3. DFT calculation 21

2.4. Kinetic simulation of relative excited state population of PCs 22

Supplementary Note 3. Mechanism study 23

3.1. Driving force evaluation 23

3.2. Proposed reaction mechanisms 23

3.3. Comparison of UV-vis absorption spectra 24

3.4. Delayed fluorescence decay quenching experiments 25

Supplementary Note 4. ATRP with photoredox/copper dual catalysis for synthesis of PMMA 29

Supplementary Note 5. Coordinates of molecular structures obtained by DFT calculation 49

Supplementary References 73

Supplementary Note 1. General information

1.1. Chemicals

4DP-IPN, 4tCz-IPN, 4Cz-IPN and 4DCDP-IPN were synthesized according to the procedures previously reported by our group.[1–3] All chemicals and solvents for the syntheses were purchased commercially (Aldrich, TCI, Alfa Aesar, and etc.) and used without further purifications. Tetrabromofluorescein (Eosin Y, TCI), tris(2-phenylpyridinato)iridium(III) (*fac*-Ir(ppy)$_3$, TCI), Perylene (TCI), Rhodamine 6G (Alfa Aesar), ethyl α-bromophenylacetate (EBPA, Alfa Aesar), copper(II) Bromide (Cu(II)Br_2, Alfa Aesar), methyl methacrylate (MMA, Aldrich), methyl acrylate (MA, Aldrich), styrene (St, Aldrich), tris(2-pyridylmethyl)amine (TPMA, TCI), *N,N*-dimethylformamide (DMF, Alfa Aesar), acetonitrile (ACN, Aldrich), dichloromethane (DCM, Aldrich), Tetrahydrofuran (THF, Samchun Chemicals) were purchased commercially. Monomers were purified by passing from basic alumina (Aldrich) to remove the inhibitors.

1.2. General experimental procedures

■ General experimental procedures for polymerization.

Polymerization, for instance, with $[MMA]_0$:$[EBPA]_0$:$[PC]_0$:$[Cu(II)Br_2]_0$:$[TPMA]_0$ = [200]:[1]:[0.0002]:[0.002]:[0.009] were typically carried out as follows. A 20 mL vial (glass, Sungho SIGMA) equipped with a stirring bar was charged with MMA (1.0 mL, 9.29 mmol), EBPA (8.39 µL, 0.046 mmol), PC (0.0093 µmol), Cu(II)Br_2 (0.093 µmol), TPMA (0.42 µmol) and anhydrous DMF (1 mL) as solvent. Pre-pared stock solution of the PCs, Cu(II)Br_2/TPMA and the initiator were used for the reproducible results. After then, the vial was capped with a rubber septum and sealed with parafilm and degassed with 99.999% N_2 for 30 min. Subsequently, polymerization was carried out under the irradiation of 455 nm (50 mW cm^{-2}) for 24 h at r.t. For the gel permeation chromatography (GPC) measurements, the in-situ aliquots were diluted in THF and purified to remove remaining Cu(II)Br_2 by passing neutral alumina to remove the Cu(II)Br_2.

■ General experimental procedures for block copolymerization.

Polymerization for PMMA macroinitiator (i.e., PMMA-Br), for instance, with $[MMA]_0$:$[EBPA]_0$:$[4DCDP\text{-}IPN]_0$:$[Cu(II)Br_2]_0$:$[TPMA]_0$ = [100]:[1]:[0.000005]:[0.001]:[0.0045] were carried out as follows. A 20 mL vial equipped with a stirring bar was charged with MMA (2.0 mL, 18.59 mmol), EBPA (33.54 µL, 0.19 mmol), PC (0.00093 µmol), Cu(II)Br_2 (0.19 µmol), TPMA (0.84 µmol) and anhydrous DMF (2 mL) as solvent. Pre-pared stock solution of the PCs, Cu(II)Br_2/TPMA and the initiator were used for the higher reproducibility results. After then, the vial was capped with a rubber septum and sealed with parafilm and bubbled with 99.999% N_2 for 30 min. Subsequently, polymerization was carried out under 455 nm irradiation (50 mW cm^{-2}) for 24 h at r.t. To isolate the macroinitiator, the reaction mixture was precipitated into containing methanol (100 mL) dropwise. Subsequent stirring for 30 min followed by vacuum filtration resulted in dried polymer which can be used as a macroinitiator (M_n = 7,500, Đ = 1.22). Subsequently, block copolymerization were carried out under the reaction conditions, for instance, with $[M]_0$:$[PMMA\text{-}Br]_0$:$[4DCDP\text{-}IPN]_0$:$[Cu(II)Br_2]_0$:$[TPMA]_0$ = [200]:[1]:[0.00001]:[0.002]:[0.009] in anhydrous DMF (2 mL, (Monomer + Macroinitiator)/DMF = 1/4 (w/v)). After then, a 20 mL vial equipped with a stirring bar was capped with a rubber septum and sealed with parafilm and bubbled with 99.999% N_2 for 30 min. Subsequently, polymerization was carried out under 455 nm irradiation (50 mW cm^{-2}) for 24 h at r.t.

■ **General experimental procedures for large-scale polymerization.**

Large-scale polymerizations, for instance, with $[MMA]_0$:$[EBPA]_0$:$[4DCDP\text{-}IPN]_0$:$[Cu(II)Br_2]_0$:$[TPMA]_0$ = [200]:[1]:[0.00001]:[0.002]:[0.009] were typically carried out as follows. A 250 mL one-neck round flask equipped with a stirring bar was charged with MMA (100 mL, 929 mmol), EBPA (0.84 mL, 4.65 mmol), PC (0.047 μmol), $Cu(II)Br_2$ (9.29 μmol), TPMA (41.83 μmol) and anhydrous DMF (100 mL) as solvent. Pre-pared stock solution of the PCs and $Cu(II)Br_2$/TPMA were used for the reproducible results. After then, the flask was capped with a rubber septum and sealed with parafilm without any degassing process. Subsequently, the polymerization was carried out under the irradiation of 455 nm (200 mW cm^{-2}) for 24 h at r.t.

■ **Experimental procedure for initial kinetic study**

Kinetic study in synthesis of PMMA with $[MMA]_0$:$[EBPA]_0$:$[PC]_0$:$[Cu(II)Br_2]_0$:$[TPMA]_0$= [200]:[1]:[0.0002]:[0.002]:[0.009] was typically carried out as follows. A 20 mL vial equipped with a stirring bar was charged with MMA (1.0 mL, 9.29 mmol), EBPA (8.39 μL, 0.046 mmol), PC (0.0093 μmol), $Cu(II)Br_2$ (0.093 μmol), TPMA (0.42 μmol) and anhydrous DMF (1 mL) as solvent. Pre-pared stock solution of the PCs, $Cu(II)Br_2$/TPMA and the initiator were used for the reproducible results. After, the vial was capped with a rubber septum and sealed with parafilm and degassed with 99.999% N_2 for 30 min. Subsequently, polymerization was carried out for 8 h under the irradiation of 455 nm (50 mW cm^{-2}) at r.t. A 0.2 mL aliquot of the reaction mixture was collected.

■ **Experimental procedure for kinetic study**

Kinetic study in synthesis of PMMA with reaction condition $[MMA]_0$:$[EBPA]_0$:$[4DCDP\text{-}IPN]_0$:$[Cu(II)Br_2]_0$:$[TPMA]_0$ = [200]:[1]:[0.00001]:[0.002]:[0.009] was typically carried out as follows. A 20 mL vial equipped with a stirring bar was charged with MMA (1.0 mL, 9.29 mmol), EBPA (8.39 μL, 0.046 mmol), PC (0.00047 μmol), $Cu(II)Br_2$ (0.093 μmol), TPMA (0.42 μmol) and anhydrous DMF (1 mL) as solvent. Pre-pared stock solution of the PCs, $Cu(II)Br_2$/TPMA and the initiator were used for the reproducible results. After, the vial was capped with a rubber septum and sealed with parafilm and degassed with 99.999% N_2 for 30 min. Subsequently, polymerization was carried out for 24 h under the irradiation of 455 nm (50 mW cm^{-2}) at r.t. A 0.2 mL aliquot of the reaction mixture was collected.

■ **Experimental procedure for temporal control**

Temporal control study of ATRP with photoredox/copper dual catalysis of MMA with reaction condition $[MMA]_0$:$[EBPA]_0$:$[4DCDP\text{-}IPN]_0$:$[Cu(II)Br_2]_0$:$[TPMA]_0$ = [200]:[1]:[0.00001]:[0.002]:[0.009] was typically carried out as follows. A 20 mL vial equipped with a stirring bar was charged with MMA (1.0 mL, 9.29 mmol), EBPA (8.39 μL, 0.046 mmol), PC (0.00047 μmol), $Cu(II)Br_2$ (0.093 μmol), TPMA (0.42 μmol) and anhydrous DMF (1 mL) as solvent. Pre-pared stock solution of the PCs, $Cu(II)Br_2$/TPMA and the initiator were used for the reproducible results. After, the vial was capped with a rubber septum and sealed with parafilm and degassed with 99.999% N_2 for 30 min. Subsequently, polymerization was carried out by switching the irradiation on for 4 h and off for 4 h. The total reaction time including both on and off steps was 20 h. A 0.1 mL aliquot of the reaction mixture was collected every irradiation on/off.

1.3 Graphical supplementary information for general procedure

■STEP 1 (stock solution) : The preparation of Cu(II)Br_2, the ligand, and PC was carried out as follows: 10.4 mg of Cu(II)Br_2, corresponding to a concentration of 5,000 ppm based on 1 mL of MMA, and 60.7 mg of TPMA, corresponding to a concentration of 22,500 ppm (4.5 molar ratio to Cu(II)Br_2), were used. PC was dissolved to achieve a concentration of 2 mg mL^{-1}, After dissolving TPMA in 10 mL of DMF, this solution was added to a vial containing Cu(II)Br_2 to prepare a Cu(II)Br_2/L total 5,000 ppm solution, dilute it tenfold to prepare a 500 ppm Cu(II)Br_2/L solution in 10 mL, and then extract 0.2 mL of this solution to achieve a concentration of 10 ppm Cu(II)Br_2/L (46.47 μM) (Supplementary Fig.1a). For PCs, start with a solution at a concentration of 100 ppm (2 mg mL^{-1}) and dilute it tenfold repeatedly until reaching a final concentration of 0.1 ppm (Supplementary Fig.1b). Since it was dissolved at a concentration of 2 mg mL^{-1}, add an amount of mL equal to half the weight of the used PC to the reaction solution (e.g., add 1.5 mL for 3 mg). When using the initiator, to achieve DP = 200, it is necessary to use 5000 ppm relative to the monomer. Therefore, a stock solution is prepared and added to reach a concentration of 23.24 mM.

■STEP 2 (sample preparation) : Prepare the reaction solution using the solvent, stock solutions (initiator, PC and Cu(II)Br_2/L), and inhibitor removed monomer, then seal it tightly with a rubber septum and parafilm (Supplementary Fig.1c). Afterward, proceed with 99.999% N_2 bubbling for 30 minutes before starting the reaction.

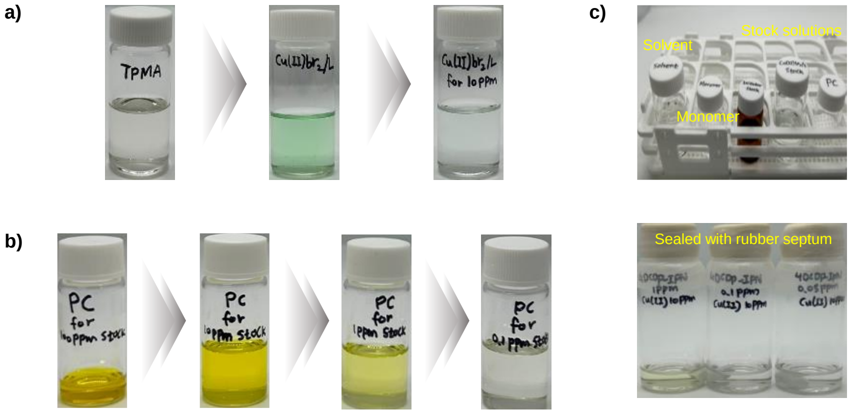

Supplementary Fig. 1 Images recorded in preparation of reaction solutions for general experimental procedures. (a) Preparation of Cu(II)Br_2/L stock solution. (b) Preparation of PC stock solution; 4DP-IPN was representatively used as a PC. (c) Images of each reagent in the reaction (upper part) and the actual reaction solution (lower part).

1.4 Instrumentation

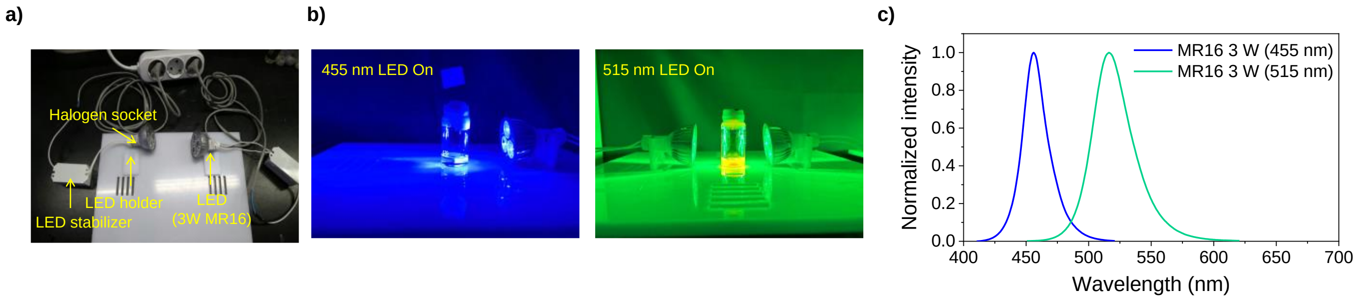

Supplementary Fig. 2 Information of reaction set-up and equipment to conduct photoreaction in this work. (a) LED set up for polymerization. (b) Using a one blue 3W MR 16 LED to achieve an irradiance of 50 mW cm^{-2} at 455 nm. For the 515 nm LED, two green 3W MR 16 LEDs were employed to achieve an irradiance of 50 mW cm^{-2}. (c) Luminescence spectra of MR 16 LEDs.

■ Photophysical measurements

UV/vis spectrophotometer at r.t. were measured with a V-770 (JASCO) UV-vis-NIR spectrometer equipped with a halogen lamp and photomultiplier tube (PMT) for a correction in UV/vis region. Steady-state photoluminescence (PL) emission spectra at r.t. were obtained with QuantaMaster 40 UV/vis steady state spectrofluorometer (Photon Technology International Inc.) equipped with a 75W Xe short arc lamp; the emission spectra were corrected for the sensitivity of the PMT. Low-temperature steady-state PL spectra was measured with a FP-8300 (JASCO) spectrofluorometer and their gated low-temperature gated PL spectra were acquired with a delay of 100 ms. PL decay measurements were carried out by the time-correlated single photon counting (TCSPC) technique on a FluoTime 200 (PicoQuant) spectrometer equipped with a PMA182 PMT (PicoQuant) using a PicoHarp 300 TCSPC board (for prompt fluorescence) and NanoHarp 250 TCSPC (for delayed fluorescence). The excitation source was a 377 nm pulsed diode laser (FWHM ~ 70 ps) (LDH series PicoQuant). The decay time fitting procedure was carried out by using the Fluofit software (PicoQuant).

■ Electrochemical measurements

Cyclic voltammetry (CV) experiments were carried out with VSP-300 Potentiostat (Bio-Logic SAS) using a one compartment electrolysis cell consisting of a glassy carbon working electrode, a Pt wire counter electrode, and a quasi Ag^+/Ag (saturated KCl aqueous solution) reference electrode (AT FRONTIER, Part No. R303). Specifically, the reference electrode is an Ag wire coated with a thin layer of AgCl and consists of a porous plug on the one end, which allow the contact between the field environment with the AgCl electrolyte. Saturated KCl aqueous solution was added inside the body of the reference electrode to stabilize the concentration of AgCl, and in this condition the electrode's reference potential is known to be +0.197 V at 25 °C. The measurements were done in 0.2 mM ACN solution with 0.1 M n-Bu_4NPF_6 (Aldrich, Electrochemical grade) as supporting electrolyte at a scan rate of 100 mV s^{-1}. All redox potentials were calibrated after each measurement against Fc^+/Fc and converted to the aqueous saturated calomel electrode (SCE) scaled by using E^0 (Fc^+/Fc) = 0.42 V vs SCE in ACN.[4] The sample solutions were degassed with 99.9999% Ar for 15 min before the measurements and then kept under the positive argon pressure during the measurements.

■ **Gel permeation chromatography (GPC) measurements**

GPC (Waters system; Waters 1515 isocratic pump, Waters 2707 autosampler) coupled with a refractive index (RI) detector (Waters 2414 RI detector), UV/vis detector (Waters 2489 UV/Vis detector), MALLS (Wyatt DAWN 8) and three different columns (Agilent Polypore 300 × 7.5 mm, Jordi mixed bed 300 × 8.0 mm, Waters Styragel HR4 300 × 7.8 mm) was used to determine the molecular weights (MWs) and dispersity (Đ) of hydrophobic polymers synthesized. THF was used as the eluent at 35 °C with a flow rate of 0.8 mL min^{-1}. PMMA standards were used for calibration of RI signal (Supplementary Fig. 3).

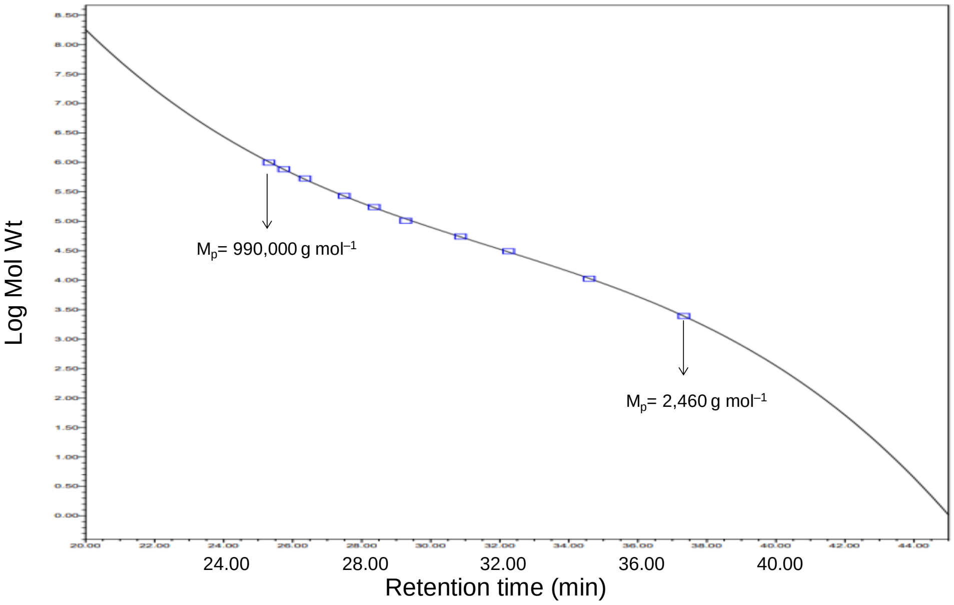

Supplementary Fig. 3 PMMA calibration curves with polymer standards M_p (peak molecular weight) (2,460, 10,600, 31,000, 55,100, 102,000, 174,000, 271,000, 525,000, 770,000, 990,000 g mol^{-1}) respectively.

1.5 Computational methods

■ **Time-dependent density functional theory (TD-DFT)**

Density functional theory (DFT) and time-dependent (TD) DFT calculations were performed with the B3LYP functional and 6–311++G* basis set as all implemented in the Gaussian16 program package (Revision C.01 x86_64 AVX-enabled Binary Version). The geometries optimization, single point energies and oscillator strengths of vertical transition were calculated in ACN solution employing the polarizable continuum model (PCM). In all geometry optimization calculations, the frequency calculations were performed both to verify that the geometries were true minima. To obtain the calculated excited state energy of PCs, vertical transition energies for S_1 and T_1 were obtained by single-point calculations on the optimized.

1.6 Synthesis of PCs

■ Synthesis of 4,4′-dicyanodiphenylamine

A solution of potassium tert-butoxide (2.22 g, 19.8 mmol), 4-aminobenzonitrile (1.95 g, 16.5 mmol) in anhydrous DMSO (30 mL) was stirred for 1 h at r.t. under a nitrogen atmosphere. After 1 h, 4-fluorobenzonitrile (2.0 g, 16.5 mmol) dissolved in DMSO (10 mL) was slowly added to the reaction mixture and stirred further at r.t. for overnight. Afterwards, distilled water was poured into the reaction mixture to quench the excess potassium tert-butoxide and to precipitate the crude product, which was further purified by reprecipitation in MeOH/water to give pure product as pale red solid (1.74 g, 48%). Data were in full agreement with those reported in literature.[2] ^{1}H NMR (400 MHz, $CDCl_3$): δ 7.62–7.57 (dt, 4H), 7.18–7.15 (dt, 4H), 6.38–7.15 (s, 1H).

■ Synthesis of 4DMDP-IPN

A solution of NaH (60% in mineral oil, 0.480 g, 12 mmol) and 4,4'-dimethoxydiphenylamine (2.017 g, 8.80 mmol) in anhydrous DMSO (8 mL) was stirred for 30 min in water bath under a nitrogen atmosphere. After 30 min, tetrafluoroisophthalonitrile (0.4 g, 2.0 mmol) dissolved in DMSO (2 mL) was slowly added to the reaction mixture and stirred further at 60 °C for 24 h. Afterwards, distilled water (2 mL) was poured into the reaction mixture to quench the excess NaH and, ethyl acetate was added to precipitate the crude product, which was further purified by column chromatography on silica gel (ethyl acetate:hexanes, 2:3 v/v) to give pure product as red solid. ^{1}H NMR data in full agreement with those reported in literature.[5] ^{1}H NMR (400 MHz, DMSO-D_6) δ 7.05–6.97 (m, 4H), 6.82–6.75 (m, 4H), 6.60 (s, 16H), 6.48 – 6.39 (m, 8H), 3.67 (d, J = 1.4 Hz, 6H), 3.59 (d, J = 1.4 Hz, 12H),3.51 (s, 6H).

■ Synthesis of 4DP-IPN

A solution of NaH (60% in mineral oil, 0.477 g, 11.94 mmol) and diphenylamine (1.48 g, 8.75 mmol) in anhydrous DMAc (5 mL) was stirred for 30 min in ice bath under a nitrogen atmosphere. After 30 min, 2,4,5,6-tetrafluoroisophthalonitrile (0.4 g, 1.99 mmol) dissolved in DMAc (5 mL) was slowly added to the reaction mixture and stirred further at 100 °C for 10 h. Afterwards, distilled water (2 mL) was poured into the reaction mixture to quench the excess NaH and, methanol was added to precipitate the crude product, which was further purified by column chromatography on silica gel (CH_2Cl_2:hexanes, 2:3 v/v) to give pure product as yellow powder (1.32 g, 83%). ^{1}H NMR data in full agreement with those reported in literature.[1,2] ^{1}H NMR (400 MHz, DMSO-d_6): δ 7.31–7.21 (m, 8H), 7.10–7.05 (t, 8H), 7.03–6.97 (t. 2H), 6.91–6.75 (m, 16H), 6.68–6.62 (m, 6H).

■ Synthesis of 4tCz-IPN

A solution of t-BuOK (0.193 g, 1.72 mmol) and 3,6-di-tert-butyl-9H-carbazole (0.400 g, 1.43 mmol) in anhydrous THF (40 mL) was stirred for 30 min in ice bath under a nitrogen atmosphere. After, 2,4,5,6- tetrafluoroisophthalonitrile (0.057 g, 0.28 mmol) was slowly added to the reaction mixture and stirred further for 12 h. After completion of the reaction, distilled water (2 mL) was poured into the reaction mixture to quench the excess NaH. The resulting solution was concentrated under reduced pressure followed by washing several times with water and ethanol to yield the crude product, which was purified by column chromatography on silica gel (CH_2Cl_2:hexanes, 2:1 v/v) to give pure product (0.160 g, 45%). Data were in full agreement with those reported in literature.[1] ^{1}H NMR (400 MHz, $CDCl_3$): δ 8.21 (d, 2H), 7.74 (dd, 2H), 7.61–7.59 (m, 6H), 7.18 (d, 2H), 7.05–7.00 (m, 8H), 6.51 (dd, 2H), 6.44 (d, 2H), 1.53 (s, 18H), 1.30 (s, 36H), 1.22 (s, 18H).

■ **Synthesis of 4Cz-IPN**

A solution of NaH (60% in mineral oil, 0.738 g, 18.45 mmol) and carbazole (2.16 g, 12.30 mmol) in anhydrous THF (40 mL) was stirred for 30 min in ice bath under a nitrogen atmosphere. After, 2,4,5,6-tetrafluoroisophthalonitrile (0.5 g, 2.46 mmol) was slowly added to the reaction mixture and stirred further for 12 h. After completion of the reaction, distilled water (2 mL) was poured into the reaction mixture to quench the excess NaH. The resulting solution was concentrated under reduced pressure followed by washing several times with water and ethanol to yield the crude product, which was purified by column chromatography on silica gel (CH_2Cl_2:hexanes, 2:1 v/v) to give pure product (1.48 g, 93%) Data were in full agreement with those reported in literature.[1,6,7] ^{1}H NMR (400 MHz, CDCl3): δ 8.22 (dt, 2H), 7.74–7.67 (m, 8H), 7.49 (ddd, 2H), 7.33 (dt, 2H), 7.23–7.21 (m, 4H), 7.12–7.05 (m, 8H), 6.82 (td, 4H), 6.63 (ddd, 2H).

■ **Synthesis of 4DCDP-IPN**

A solution of NaH (60% in mineral oil, 0.120 g, 3 mmol) and 4,4′-dicyanodiphenylamine (0.548 g, 2.5 mmol) in anhydrous DMAc (5 mL) was stirred for 30 min in ice bath under a nitrogen atmosphere. After 30 min, 2,4,5,6-tetrafluoroisophthalonitrile (0.1 g, 0.5 mmol) dissolved in DMAc (5 mL) was slowly added to the reaction mixture and stirred further at 60 ℃ for 12 h. Afterwards, distilled water (2 mL) was poured into the reaction mixture to quench the excess NaH and, methanol was added to precipitate the crude product, which was further purified by reprecipitation in CH_2Cl_2/ethyl acetate to give pure product as yellow solid (0.140 g, 28%). Data were in full agreement with those reported in literature.[2] ^{1}H NMR (400 MHz, acetone-d_6): δ 7.87–7.81 (d, 4H), 7.70–7.62 (d, 8H), 7.55–7.48 (td. 8H), 7.20–7.15 (d, 8H), 7.10–7.03 (d, 4H)

Supplementary Note 2. Characterization of PCs

2.1. Previously reported ATRP with photoredox/copper dual catalysis

Supplementary Table 1 Summary of previously reported ATRP with photoredox/copper dual catalysis.

Year	PC	Chemical structure	Polymerization condition	Reference
2013	ZnO	-	Light irradiation : 350 nm<br>$Cu(II)Br_2$: 5,000 ppm<br>PC : 5,000 ppm<br>Monomer : MMA	(8)
2014	mpg-C_3N_4	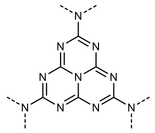	Light irradiation : 350 nm and sunlight<br>$Cu(II)Br_2$: 5,000 ppm<br>PC : 8 mg mL^{-1}<br>Monomer : MMA, MA and St	(9)
2018	Polymethine	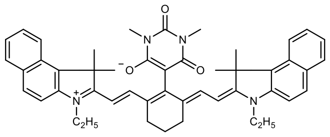	Light irradiation : 790 nm<br>$Cu(II)Br_2$: 100 ppm<br>PC : 1,000 ppm<br>Monomer : MMA	(10)
2020	Carbon dots	(Sodium alginate + ethylene diamine)	Light irradiation : 405 nm<br>$Cu(II)Br_2$: 100 ppm<br>PC : 1,000 ppm<br>Monomer : MMA	(11)
2020	UCNPs	-	Light irradiation : 980 nm<br>$Cu(II)Br_2$: 100 ppm<br>PC : 4.8 mg mL^{-1}<br>Monomer : MA	(12)
2021	TFPPy-Td-COFs	-	Light irradiation : white LED<br>$Cu(II)Br_2$: 1,000 ppm<br>PC : 4 mg mL^{-1}<br>Monomer : MMA	(13)
2021	PTZ-CMP	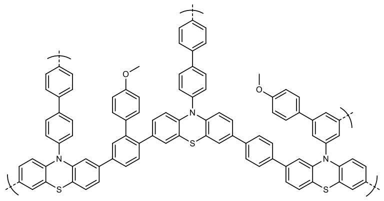	Light irradiation : 520 and 660 nm<br>$Cu(II)Br_2$: 200 ppm<br>PC : 2 mg mL^{-1}<br>Monomer : MA	(14)
2021	*ht*-PLP_{PAN}	-	Light irradiation : 450 and 520 nm<br>$Cu(II)Br_2$: 500 ppm<br>PC : 0.5 mg mL^{-1}<br>Monomer : $OEGMA_{500}$	(15)
2022	ZnPor	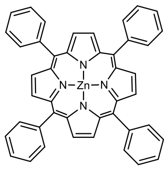	Light irradiation : 630 nm<br>$Cu(II)Br_2$: 200 ppm<br>PC : 5 or 10 ppm<br>Monomer : MA	(16)
2022	ZnPC	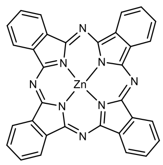	Light irradiation : 630 nm<br>$Cu(II)Br_2$: 200 ppm<br>PC : 5 or 10 ppm<br>Monomer : MA	(16)

2022	Carbon quantum dots	-	Light irradiation : 460, 530 nm and sunlight<br>Cu(II)Br$_2$: 300 ppm<br>PC : 0.25 mg mL^{-1}<br>Monomer : HEA	(17)
2022	P-CDs	-	Light irradiation : 405 nm<br>Cu(II)Br$_2$: 100 ppm<br>PC : 0.75 mg mL^{-1}<br>Monomer : MMA and St	(18)
2022	Eosin Y	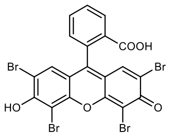	Light irradiation : 520 nm<br>Cu(II)Br$_2$: 1,000 ppm<br>PC : 50 ppm<br>Monomer : OEOMA$_{500}$	(19)
2023	Eosin Y	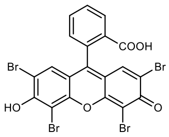	Light irradiation : 520 nm<br>Cu(II)Br$_2$: 1,000 ppm<br>PC : 50 ppm<br>Monomer : OEOMA$_{500}$<br>(Hyperbranched polymer)	(20)
2023	Eosin Y	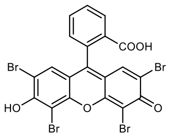	Light irradiation : 520 nm<br>Cu(II)Br$_2$: 1,000 ppm<br>PC : 50 ppm<br>Monomer : OEOA$_{480}$	(21)
2023	PPh_3-CHCP	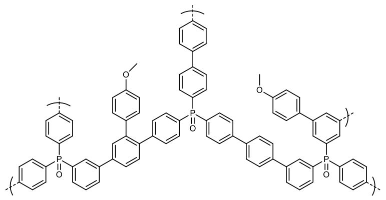	Light irradiation : 455–940 nm and sunlight<br>Cu(II)Br$_2$: 200 ppm<br>PC : 0.5 mg mL^{-1}<br>Monomer : MA, MMA and St	(22)
2023	Terephthalate	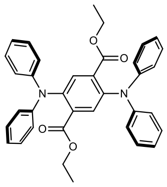	Light irradiation : 425 nm<br>Cu(II)Br$_2$: 5 ppm<br>PC : 1 ppm<br>Monomer : MMA	(23)
2023	4Cz-IPN	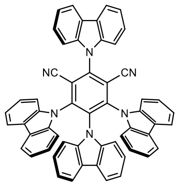	Light irradiation : 425 nm<br>Cu(II)Br$_2$: 10 ppm<br>PC : 1 ppm<br>Monomer : MMA	(23)
2023	Methylene blue	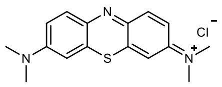	Light irradiation : 395–730 nm<br>sunlight<br>Cu(II)Br$_2$: 250–1,500 ppm<br>PC : 125 ppm<br>Monomer : OEOMA$_{500}$	(24)
2024	4DCDP-IPN	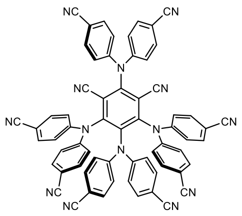	Light irradiation : 455 nm<br>Cu(II)Br$_2$: 10 ppm<br>PC : 50 ppb<br>Monomer : MMA	This work

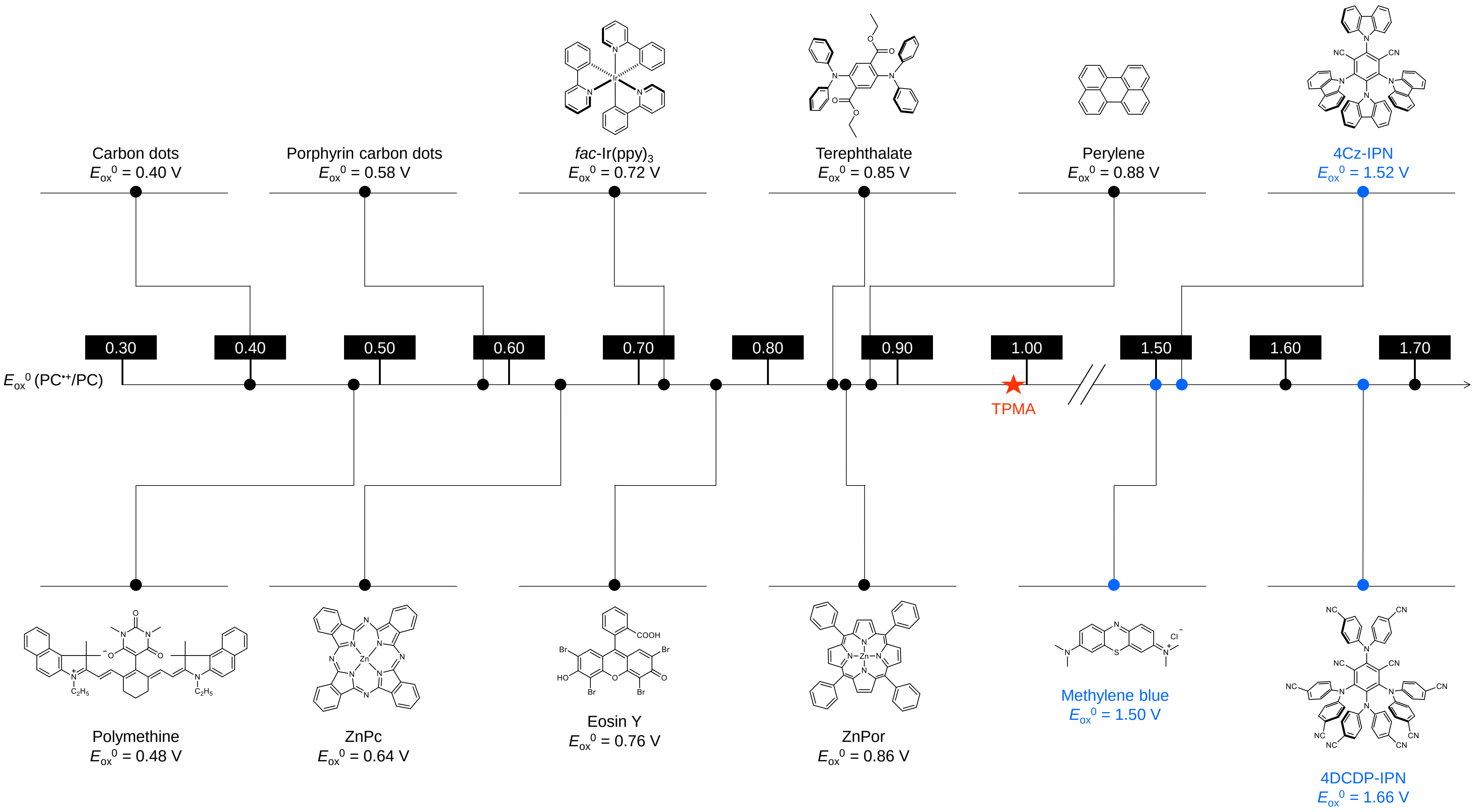

Supplementary Fig. 4 Ground state oxidation potential (E_{ox}^0 (PC$^{•+}$/PC)) of previously reported PCs in ATRP with dual photoredox/copper catalysis referred to Supplementary Table1. Their E_{ox}^0 (PC$^{•+}$/PC) were measured against SCE.

2.2. Properties of PCs

Supplementary Table 2 Photophysical properties of PCs in ACN. The photophysical rate constants were derived by experimental values for τ_{prompt}, $\tau_{delayed}$ and Φ_F (all given in Supplementary Fig. 5, Supplementary Fig. 6 and Supplementary Table 4). In this study, photophysical rate constants were determined following the procedure investigated by our group studied[2,3] under assumptions, i) $k_{nr,T1}$, $k_{r,T1}$ << k_{RISC} (i.e., $k_{nr,T1}$ ~ 0 and $k_{r,T1}$ ~ 0) and ii) k_{ISC} >> k_{RISC}.

PC	E_{00} (S$_1$) (eV)[a]	E_{00} (T$_1$) (eV)[b]	τ_{prompt} (ns)	$\tau_{delayed}$ (μs)	$\lambda_{max,abs.}$ (nm)	$\lambda_{max,em.}$ (nm)	$f_{S0\rightarrow S1}$ [c]	Φ_F	$k_{r,S1}$ (10^7 s^{-1})[d]	$k_{nr,S1}$ (10^7 s^{-1})	k_{ISC} (10^8 s^{-1})	$k_{r,T1}$ (s^{-1})	$k_{nr,T1}$ (10^3 s^{-1})	k_{RISC} (10^5 s^{-1})
4DMDP-IPN	2.38	2.24	1.4	4.2	493	586	0.0636	0.01	1.9	180	–[e]	–	–	0.94
4DP-IPN	2.45	2.29	3.4	121.5	469	531	0.0790	0.61	2.96	1.90	2.49	–	–	5.04
4tCz-IPN	2.61	2.61	8.3	1.3	440	578	0.0863	0.05	2.4	45	–[e]	–	–	2.0
4Cz-IPN	2.65	2.61	13.6	1.8	420	551	0.0697	0.18	0.55[f]	4.60[f]	0.022[f]	–	–	1400[f]
4DCDP-IPN[g]	2.58	2.41	1.8	196.1	458	500	0.0964	0.61	4.2	2.7	4.9	–	–	0.41

[a,b]E_{00} were extracted from the [a]onset of PL in ACN at r.t. and [b]onset of gated PL in ACN at 65 K, respectively. The onsets were obtained by tangential method; i.e., the intersection of the tangent, set at the high energy slope of the spectrum, with the x-axis.[25] [c]Oscillator strengths were obtained by TD-DFT calculation. [d]$k_{r,S1}$ was estimated via the Strickler-Berg equation.[26,27] [e]Because of i) the approximation conditions and/or ii) the error in the experimental and theoretical (Strickler-Berg equation) determination of k_{ISC}, the rate constants were evaluated as negative values. [f]Photophysical properties of 4Cz-IPN in ACN were referred from the literature.[28] [g]Photophysical properties of 4DCDP-IPN in ACN were referred to the literature.[2]

Supplementary Table 3 Summarized redox potentials of PCs in ACN.

PC	E_{red}^0	E_{ox}^0	$E_{red}^*(S_1)$	$E_{red}^*(T_1)$	$E_{ox}^*(S_1)$	$E_{ox}^*(T_1)$
4DMDP-IPN	–1.70	0.76	0.68	0.54	–1.62	–1.48
4DP-IPN	–1.66	1.01	0.79	0.63	–1.44	–1.28
4tCz-IPN	–1.31	1.30	1.30	1.30	–1.31	–1.31
4Cz-IPN	–1.21	1.52	1.44	1.40	–1.13	–1.09
4DCDP-IPN	–1.13	1.66	1.45	1.28	–0.92	–0.75

Excited state redox potentials of PCs were calculated by Rehm-Weller equation,[29] $E_{red}^*(S_1)$ (V vs SCE) = E_{red}^0 (V vs SCE) + E_{00} (S_1) (eV), $E_{red}^*(T_1)$ (V vs SCE) = E_{red}^0 (V vs SCE) + E_{00} (T_1) (eV), $E_{ox}^*(S_1)$ (V vs SCE) = E_{ox}^0 (V vs SCE) - E_{00} (S_1) (eV) and $E_{ox}^*(T_1)$ (V vs SCE) = E_{ox}^0 (V vs SCE) - E_{00} (T_1) (eV).

Supplementary Table 4 Photoluminescence quantum yield (Φ_F) of PCs.

PC	Solvent	Reference	Φ_F	
			Degassed with N_2	Air
4DMDP-IPN[a]	ACN	Coumarin 153[e]	0.01	0.01
4DP-IPN[b]	ACN	–[c]	0.69	0.10
		Fluorescein[d]	0.63	0.08
		Coumarin 153[e]	0.61	0.07
4tCz-IPN[b]	ACN	Coumarin 153[e]	0.05	0.04
4Cz-IPN[b]	ACN	Fluorescein[d]	0.20	0.14
		Coumarin 153[e]	0.18	0.12
4DCDP-IPN[b]	ACN	Coumarin 153[e]	0.61	0.16

[a]Photoluminescence quantum yield values were measured in this work. [b]Photoluminescence quantum yield values are referred to literature. [c]Absolute PLQY measurement. Relative PLQY measurement against [d]fluorescein (1.0×10^{-5} M) in 0.1 M NaOH aqueous solution ($\Phi_F = 0.824 \pm 0.049$) or [e]coumarin 153 (1.0×10^{-5} M) in ethanol solution ($\Phi_F = 0.514 \pm 0.031$).[30]

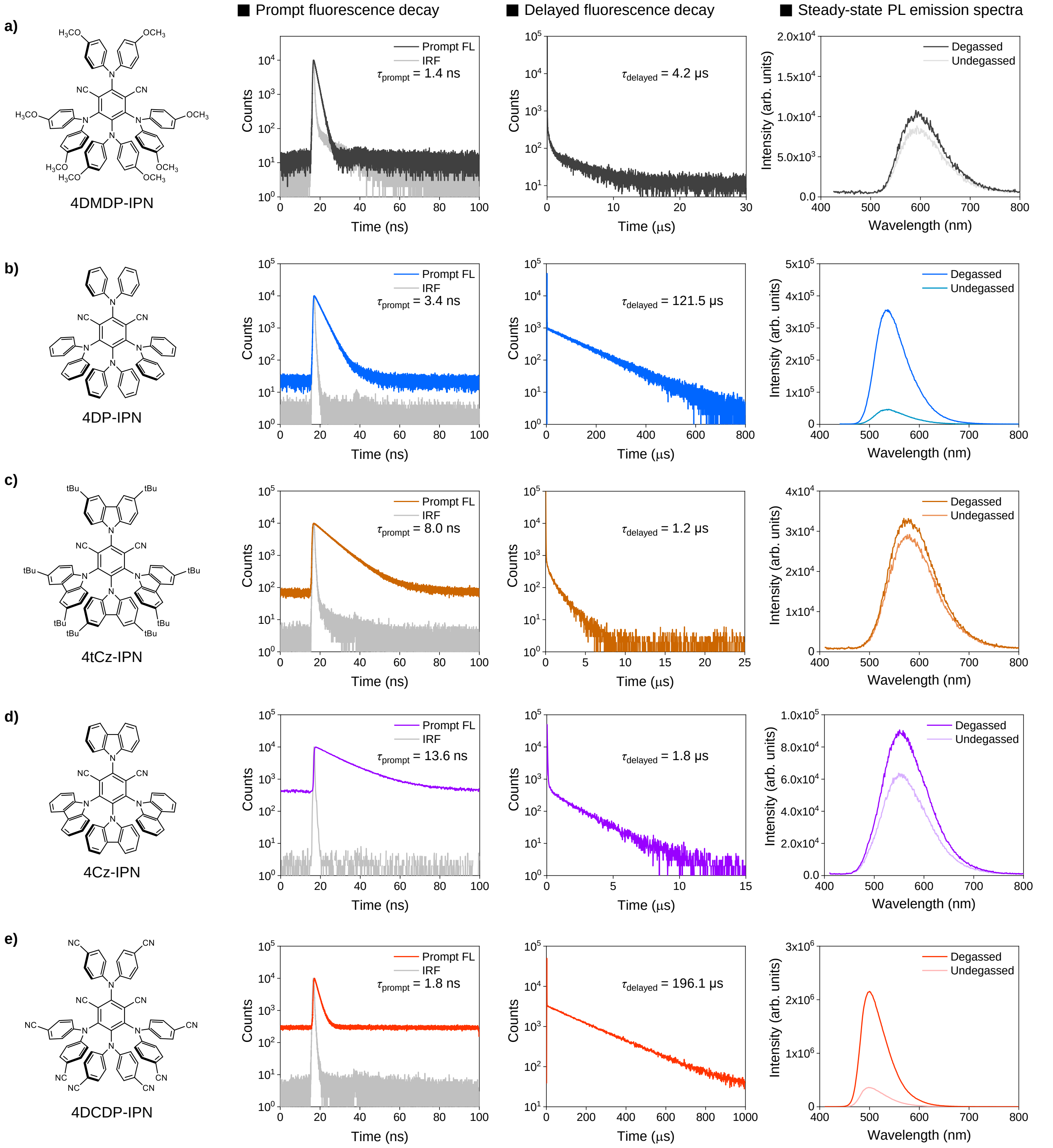

Supplementary Fig. 5 Chemical structures, prompt fluorescence decay (left), delayed fluorescence decay (center) and steady-state PL emission spectra (right) of (a) 4DMDP-IPN, (b) 4DP-IPN, (c) 4tCz-IPN, (d) 4Cz-IPN and (e) 4DCDP-IPN. Generally, prompt and delayed fluorescence decay spectra were taken from the undegassed and degassed solutions of PCs (1.0×10^{-5} M) in ACN at r.t., respectively.

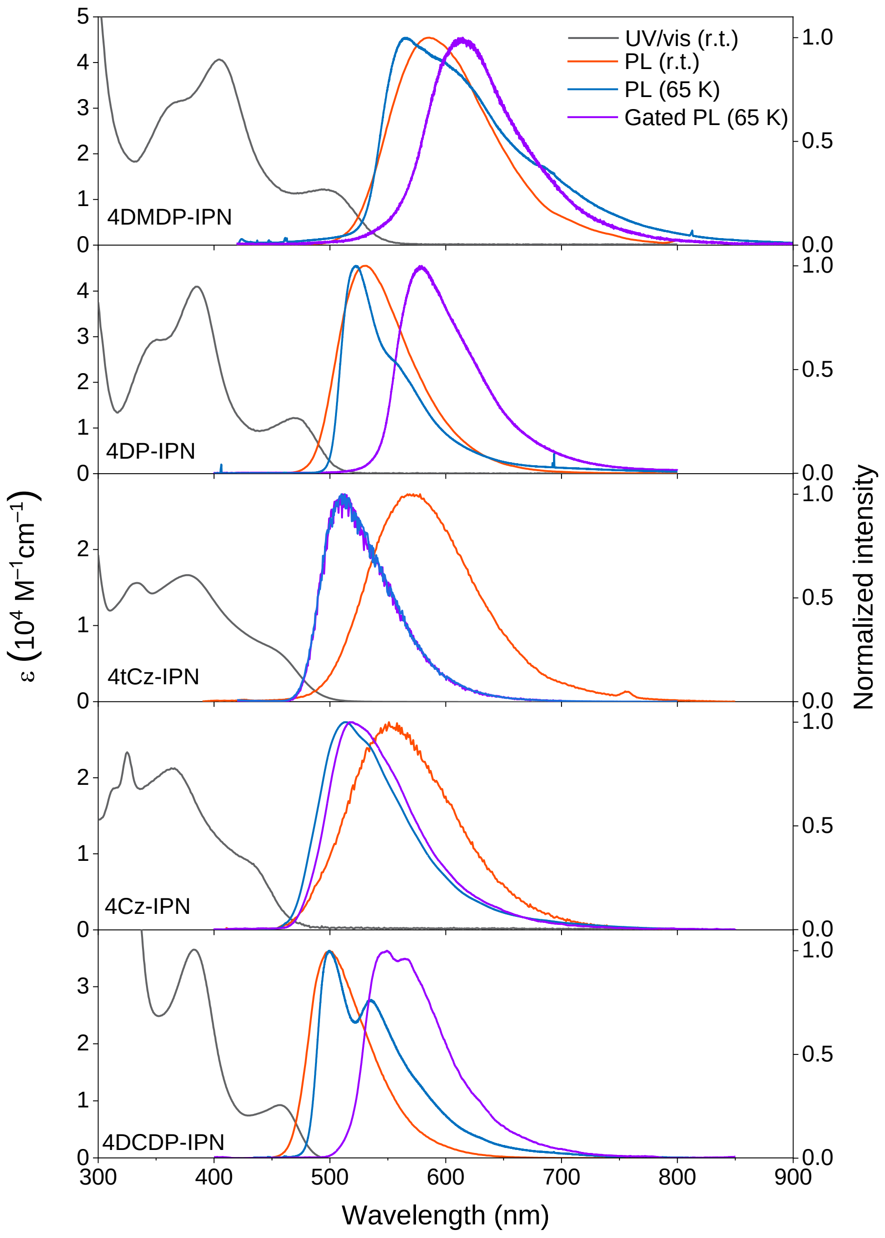

Supplementary Fig. 6 UV/vis absorption and PL emission spectra of PCs. UV/vis absorption spectra of PC (1.0×10^{-5} M) in ACN solution were measured at r.t. Steady-state PL emission spectra of PCs (1.0×10^{-5} M) in ACN at r.t. and 65 K. Gated PL spectra at 65 K were acquired with a delay of 100 ms.

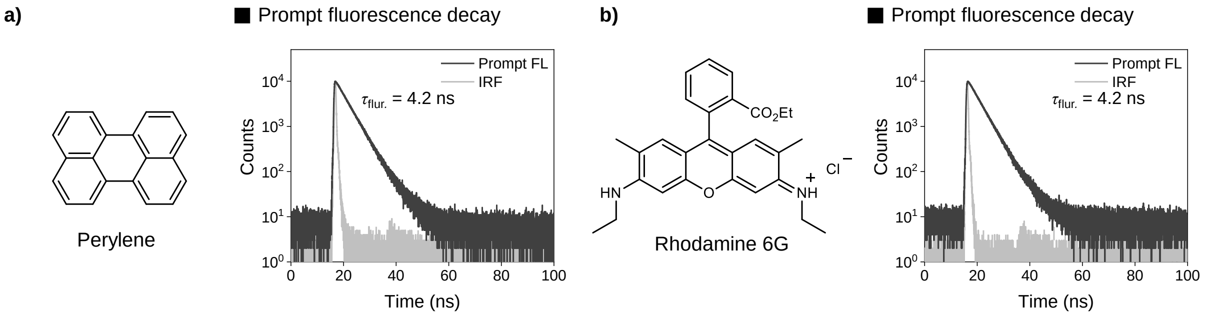

Supplementary Fig. 7 Chemical structures and prompt fluorescence decay of (a) perylene and (b) Rhodamine 6G. Generally, prompt fluorescence decay at r.t. spectra were taken from the undegassed solutions of PCs (1.0×10^{-5} M) in ACN at r.t.

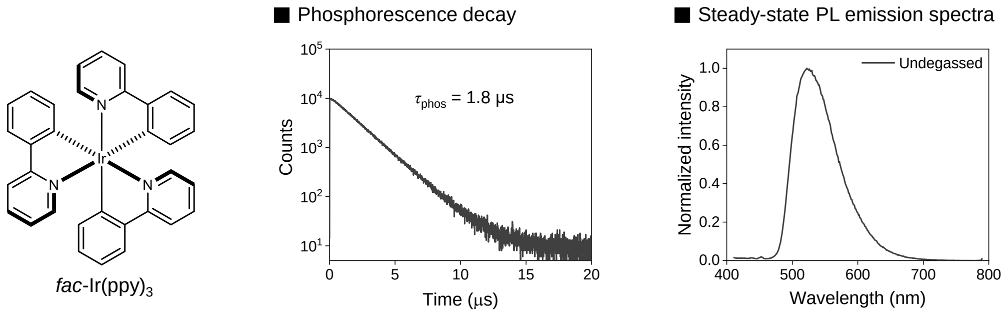

Supplementary Fig. 8 Chemical structures, Phosphorescence decay (left), steady-state PL emission spectra (right) of *fac*-Ir(ppy)$_3$ Generally, phosphorescence decay spectra was taken from the degassed solutions of PCs (1.0×10^{-5} M) in ACN at r.t. In case of steady-state PL emission spectra was taken from the undegassed solutions of PCs (1.0×10^{-5} M) in ACN at r.t.

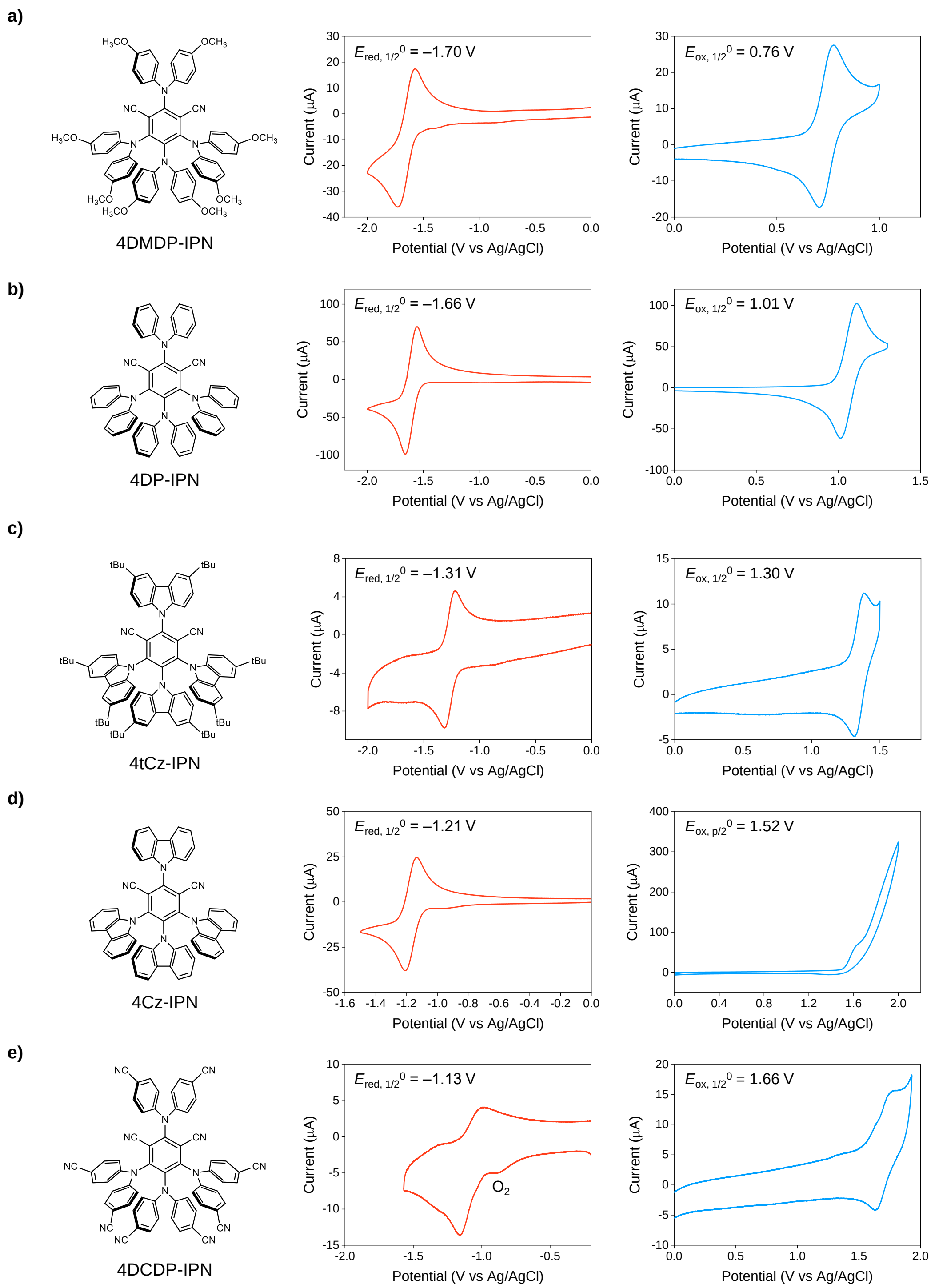

Supplementary Fig. 9 Chemical structures, CV spectra of reduction cycle (left), oxidation cycle (right) of PCs. (a) 4DMDP-IPN, (b) 4DP-IPN, (c) 4tCz-PN, (d) 4Cz-IPN and (e) 4DCDP-IPN. CV spectra of reduction cycle of PC (2.0 × 10^{-4} M) were obtained in ACN at r.t. after the degassing process by purging with Ar for 15 min. For reversible CV cycle, the potentials were evaluated as their half-potentials ($E_{1/2}^{0}$), however, for irreversible CV cycle, the potentials were obtained from their half-peak potentials ($E_{p/2}^{0}$).[4] all potentials were converted to the SCE scale by using E^{0} (Fc^{+}/Fc) = 0.42 V vs SCE in ACN.[4]

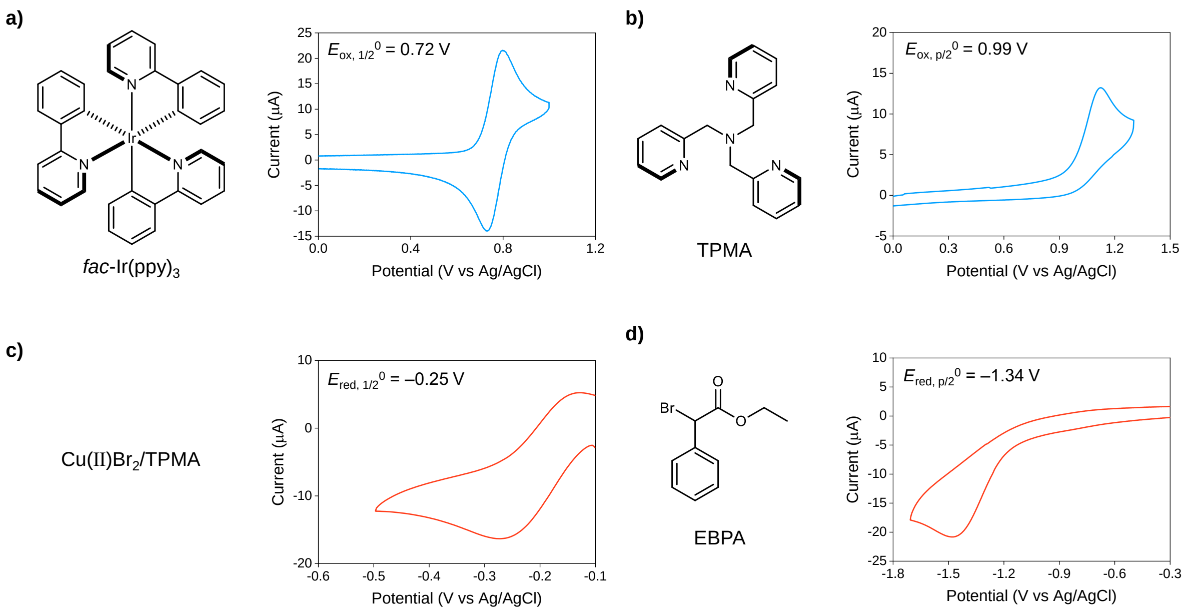

Supplementary Fig. 10 Chemical structures and CV spectra of PC and ATRP components studied in this work. (a) *fac*-Ir(ppy)$_3$, (b) TPMA, (c) Cu(II)Br$_2$/TPMA ([Cu(II)Br$_2$]$_0$:[TPMA]$_0$ = (molar ratio = 1:1)) and (d) EBPA. CV spectra were obtained in ACN (2.0 × 10^{-4} M) at r.t. after the degassing process by purging with Ar for 15 min. For irreversible CV cycle, the potentials were obtained from their half-peak potentials ($E_{p/2}^{0}$).[4] All potentials were converted to the SCE scale by using E^0 (Fc^+/Fc) = 0.42 V vs SCE in ACN.[4]

2.3. DFT calculation

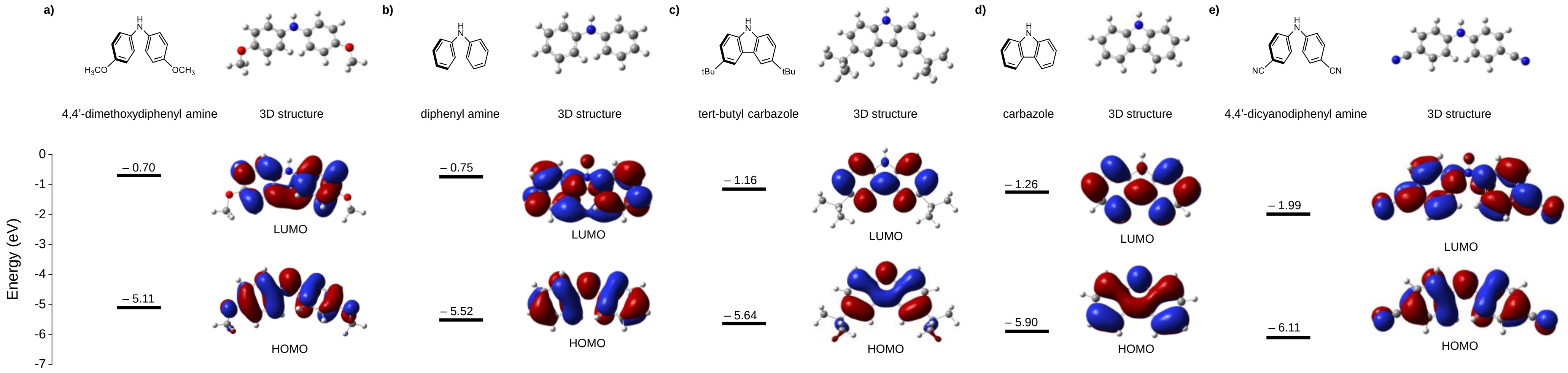

Supplementary Fig. 11 Chemical structure, optimized 3D structure, and the HOMO/LUMO energy levels obtained DFT calculation of (a) 4,4'-dimethoxydiphenyl amine, (b) diphenyl amine, (c) tert-butyl carbazole, (d) carbazole and (e) 4,4'-dicyanodiphenyl amine. All DFT calculations were performed with the B3LYP functional and 6–311++G* basis set in ACN solution employing the PCM solvation model.

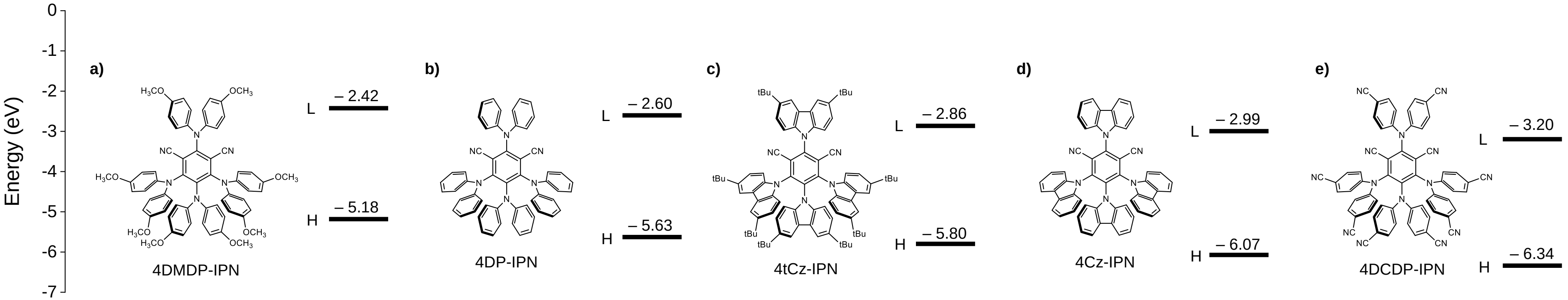

Supplementary Fig. 12 Chemical structure, and the HOMO/LUMO energy levels obtained DFT calculation of (a) 4DMDP-IPN, (b) 4DP-IPN, (c) 4tCz-IPN, (d) 4Cz-IPN and (e) 4DCDP-IPN. All DFT calculations were performed with the B3LYP functional and 6–311++G* basis set in ACN solution employing the PCM solvation model.

2.4. Kinetic simulation of relative excited state population of PCs

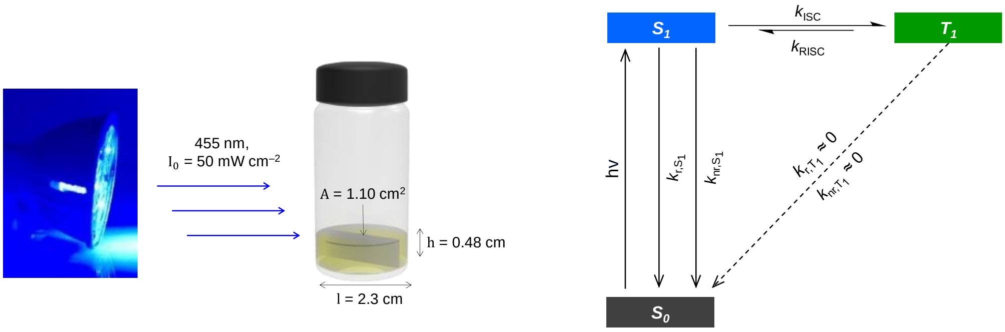

Supplementary Fig. 13 Schematic illustration of light irradiation and Jablonski diagram of PCs.

Kinetic simulation were performed with the ordinary differential equations (ODE) based on the rate law using ODE 15s solver implemented in the Matlab program package (R2024a version).[2,31] The photophysical behaviors of S_1 and T_1 are depicted in Supplementary Fig. 13. Thus, the concentration of S_1 and T_1 can be described by following system of ordinary differential equations (ODE).

$$\frac{d[S_1]}{dt} = P + k_{RISC}[T_1] - (k_{ISC} + k_{r,S_1} + k_{nr,S_1})[S_1] \quad (1)$$

$$\frac{d[T_1]}{dt} = k_{\mathrm{ISC}}[S_1] - (k_{\mathrm{RISC}} + k_{\mathrm{r,T_1}} + k_{\mathrm{nr,T_1}})[T_1] \quad (2)$$

where P is a rate of S_1 generation from S_0 via photoexcitation and k_{ISC}, k_{RISC}, $k_{r,S1}$, $k_{r,T1}$, $k_{nr,S1}$ and $k_{nr,T1}$ denote the rate constants of intersystem crossing, reverse-intersystem crossing, radiative decay from S_1, radiative decay from T_1, non-radiative decay from S_1, and non-radiative decay from T_1, respectively. P can be expressed by following equation,

$$P = \phi \times \frac{A}{V_0 N_A} \times \frac{I_0}{h\nu} \times (1 - 10^{-\varepsilon c l}) \quad (3)$$

where ϕ is quantum yield of the transformation from $S_0 \rightarrow S_1$, A is cross-sectional area (1.10 cm^2), V_0 is the reaction volume (2 mL), N_A is Avogadro number (6.022 x 10^{23} mol^{-1}), h is Planck constant (6.626 x 10^{-34} m^2 kg s^{-1}), ν is frequency of the photon (6.593 x 10^{14} s^{-1} (455 nm)), ε is the extinction coefficient of PCs (e.g., 1.07 x 10^4 M^{-1}cm^{-1} for 4DP-IPN), c is the concentration of [S_0], l is the optical path length (2.3 cm) and I_0 is light intensity (I_0 ~ 50 mW cm^{-2}, 455 nm). Because it is tricky to evaluate all the factors affecting photonflux such as a vial's curvature/refractive index, they are not involved in the simulation. It is assumed ϕ is unity, therefore, in accordance with our experimental conditions, P would be converted to the following equation.

$$P = 1.05 \times 10^{-4}\ \mathrm{M\ s^{-1}} \times (1 - 10^{-\varepsilon c l}) \quad (4)$$

Using the derived rate constant of photoexcitation, we conducted the kinetic simulation based on the photophysical values of PCs to solve the ODEs listed in Supplementary Table 2.

Supplementary Note 3. Mechanism study

3.1. Driving force evaluation

Supplementary Table 5 Experimental evaluation of driving forces ($-\Delta G_{ET}$) for ET process.

$-\Delta G_{ET}$ (eV)	4DMDP-IPN		4DP-IPN		4tCz-IPN		4Cz-IPN		4DCDP-IPN	
	$^1PC^*$	$^3PC^*$	$^1PC^*$	$^3PC^*$	$^1PC^*$	$^3PC^*$	$^1PC^*$	$^3PC^*$	$^1PC^*$	$^3PC^*$
PC^* + Cu(II)Br_2/TPMA → $PC^{\bullet+}$ + Cu(I)Br/TPMA + Br^-	1.37	1.23	1.19	1.03	1.06	1.06	0.88	0.84	0.67	0.50
PC^* + R-Br → $PC^{\bullet+}$ + $R^\bullet$ + Br^- (R-Br = EBPA)	0.28	0.14	0.10	–0.06	–0.03	–0.03	–0.21	–0.25	–0.42	–0.59
PC^* + TPMA → $PC^{\bullet-}$ + $TPMA^{\bullet+}$	–0.31	–0.45	–0.20	–0.36	0.31	0.31	0.45	0.41	0.46	0.29
$PC^{\bullet+}$ + TPMA → PC + $TPMA^{\bullet+}$	–0.23		0.02		0.31		0.53		0.67	
$PC^{\bullet+}$ + Cu(I)Br/TPMA + Br^- → PC + Cu(II)Br_2/TPMA	1.01		1.26		1.55		1.77		1.91	
$PC^{\bullet+}$ + $R^\bullet$ + Br^- → PC + R-Br	2.10		2.35		2.64		2.86		3.00	

The driving forces for each ET process were calculated using the following equations: $-\Delta G_{ET} = E_{red}^*(PC^*/PC^{\bullet-}) - E_{ox}^0(Sub^{\bullet+}/Sub)$ and $-\Delta G_{ET} = E_{red}^0(Sub/Sub^{\bullet-}) - E_{ox}^*(PC^{\bullet+}/PC^*)$ for photoinduced ET process; $-\Delta G_{ET} = E_{red}^0(Sub/Sub^{\bullet-}) - E_{ox}^0(PC^{\bullet+}/PC)$ for the ET process in PC regeneration.[29] Ground- and excited redox potentials of all PCs were referred from Supplementary Table 3. The ground state redox potentials of ATRP components were estimated as E_{ox}^0 = 0.99 V for TPMA, E_{red}^0 = –0.25 V for Cu(II)Br_2/TPMA, and E_{red}^0 = –1.34 V for EBPA.

3.2. Proposed reaction mechanisms

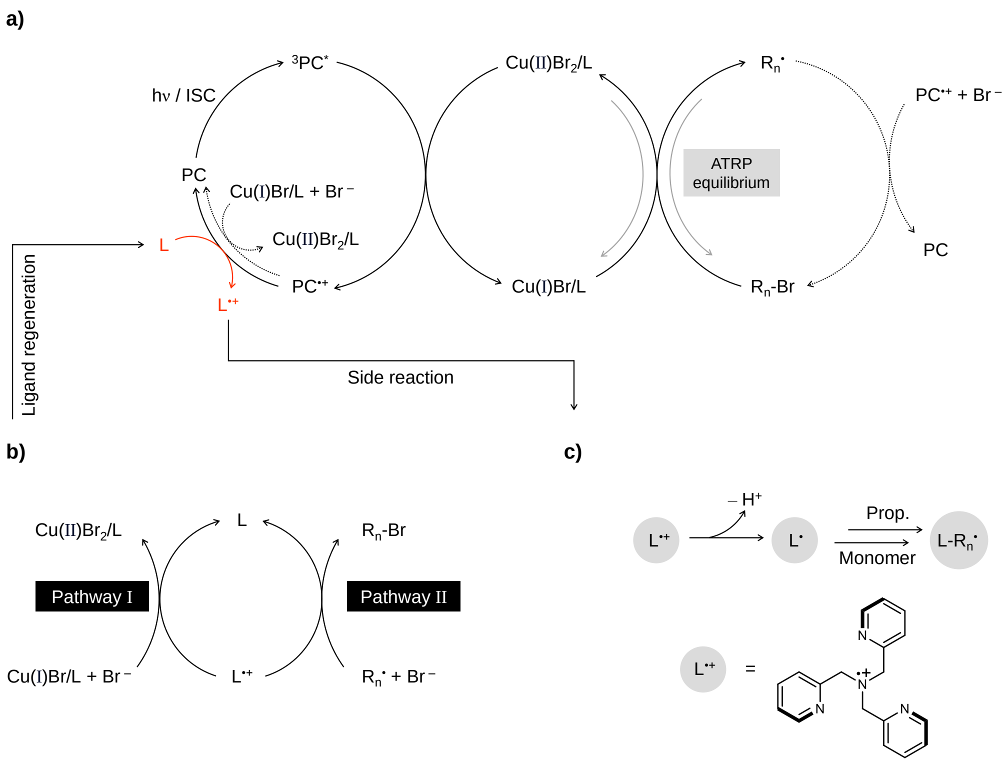

Supplementary Fig. 14 Proposed reaction mechanisms including reaction pathways of (a) the ATRP with dual photoredox/copper catalysis, (b) a ligand regeneration and (c) a side reaction from ligand radical cation ($L^{\bullet+}$).

3.3. Comparison of UV-vis absorption spectra

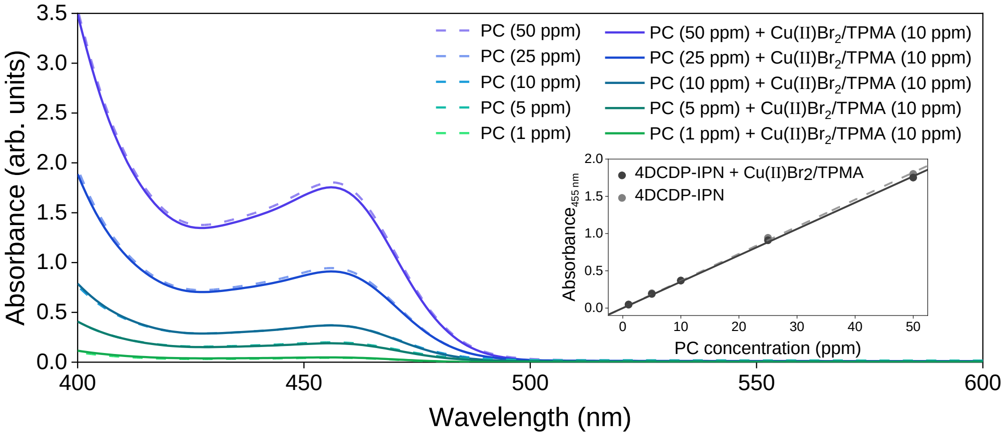

Supplementary Fig. 15 UV-vis absorption spectra of PC solutions varying concentrations in the presence of Cu(II)Br_2/TPMA. PC solutions were prepared by diluted 4DCDP-IPN solutions in DMF as 50 ppm (relative to monomer mimicking our reaction conditions), 25 ppm, 10 ppm, 5 ppm, and 1 ppm correspond to 230 µM, 115 µM, 46 µM, 23 µM, and 4.6 µM respectively. To prepare Cu(II)Br_2/TPMA solution, 1:1 molar ratio of Cu(II)Br_2 and TPMA were used. The correlation between absorbance and PC concentration with (dark solid line) or without (grey dash line) Cu(II)Br_2/TPMA were also given (inset).

3.4. Delayed fluorescence decay quenching experiments

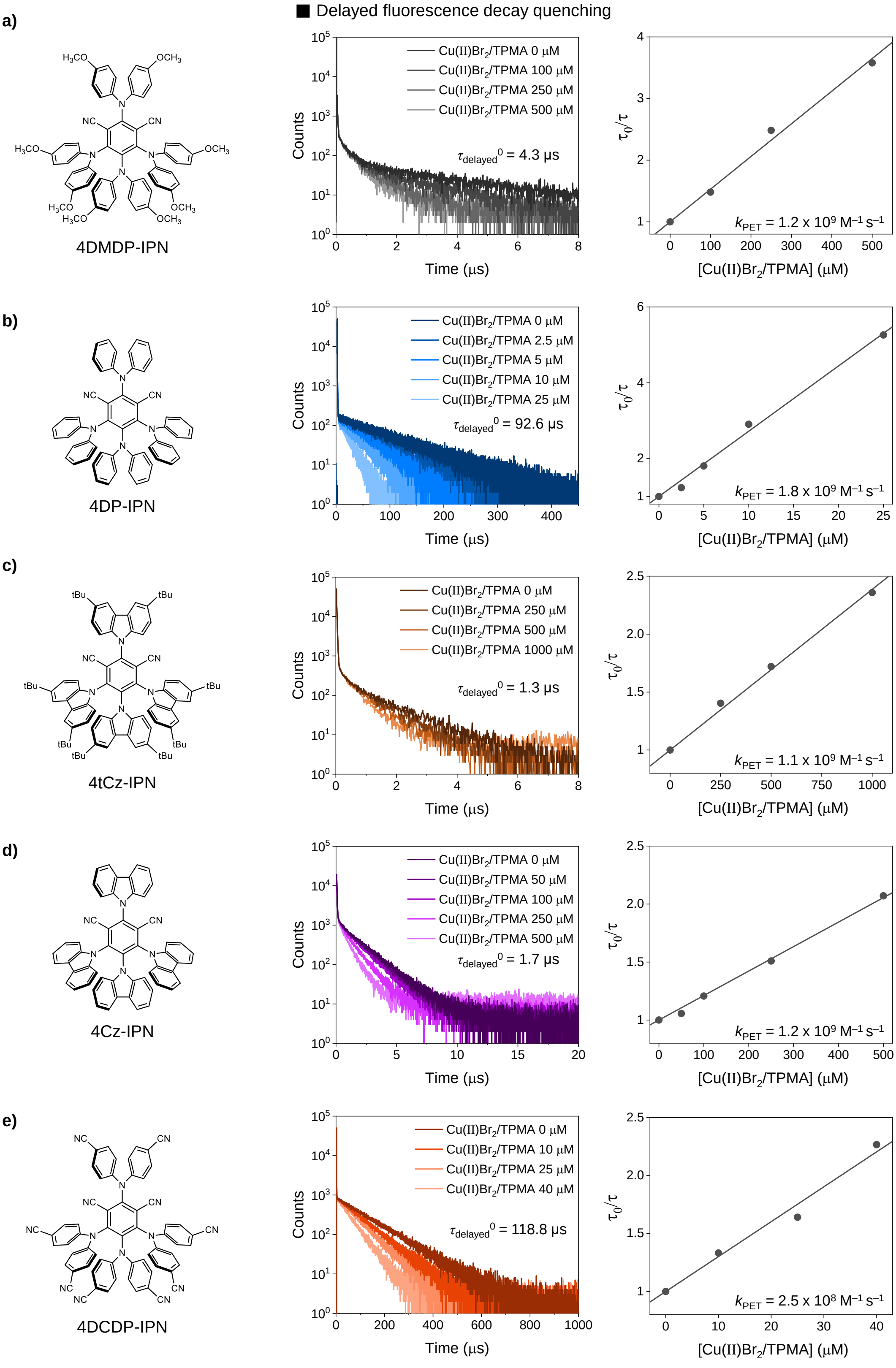

Supplementary Fig. 16 Stern-Volmer plots of PCs along with addition of Cu(II)Br$_2$/TPMA. (a) 4DMDP-IPN, (b) 4DP-IPN, (c) 4tCz-PN, (d) 4Cz-IPN and (e) 4DCDP-IPN. To avoid quenching by TPMA, it was added as equivalent to Cu(II)Br$_2$. Generally, the changes of delayed fluorescence decay were monitored using the degassed solutions of PCs (1.0×10^{-5} M) in DMF at r.t. using TCSPC technique at $\lambda_{ex.}$ = 377 nm.

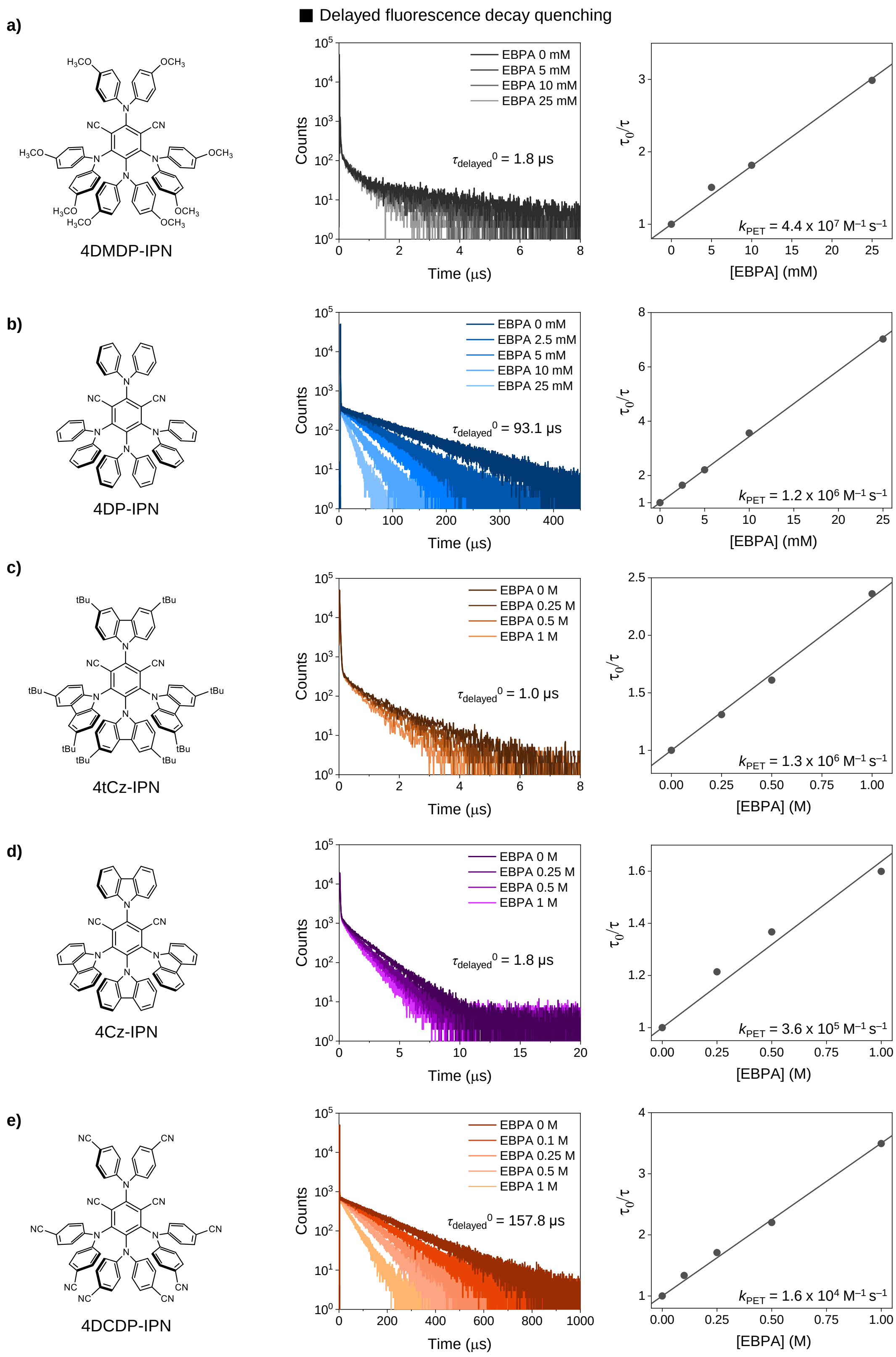

Supplementary Fig. 17 Stern-Volmer plots of PCs along with addition of EBPA. (a) 4DMDP-IPN, (b) 4DP-IPN, (c) 4tCz-PN, (d) 4Cz-IPN and (e) 4DCDP-IPN. Generally, the changes of delayed fluorescence decay were monitored using the degassed solutions of PCs (1.0×10^{-5} M) in DMF at r.t. using TCSPC technique at $\lambda_{ex.}$ = 377 nm.

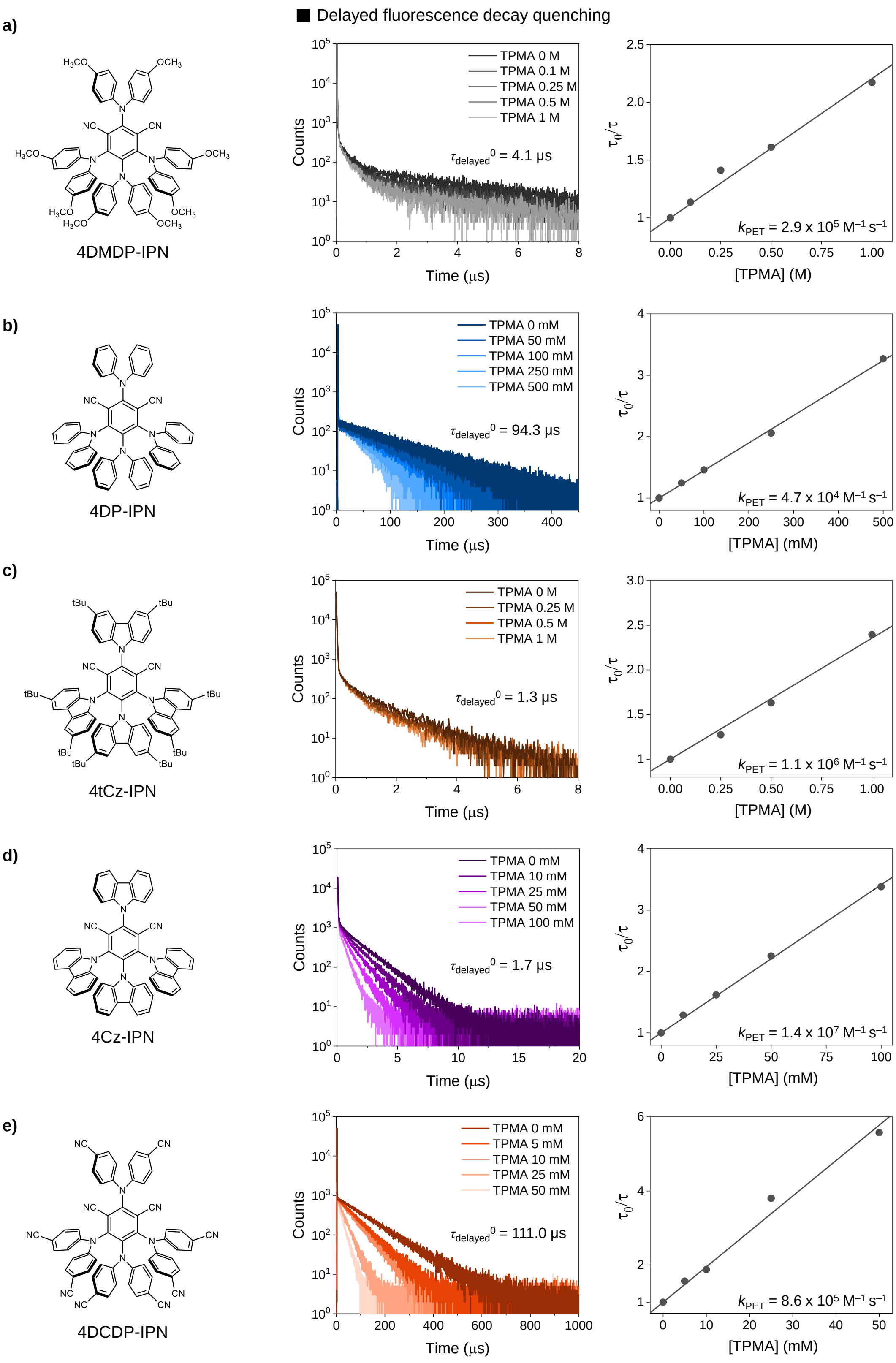

Supplementary Fig. 18 Stern-Volmer plots of PCs along with addition of TPMA. (a) 4DMDP-IPN, (b) 4DP-IPN, (c) 4tCz-PN, (d) 4Cz-IPN and (e) 4DCDP-IPN. Generally, the changes of delayed fluorescence decay were monitored using the degassed solutions of PCs (1.0×10^{-5} M) in DMF at r.t. using TCSPC technique at $\lambda_{ex.}$ = 377 nm.

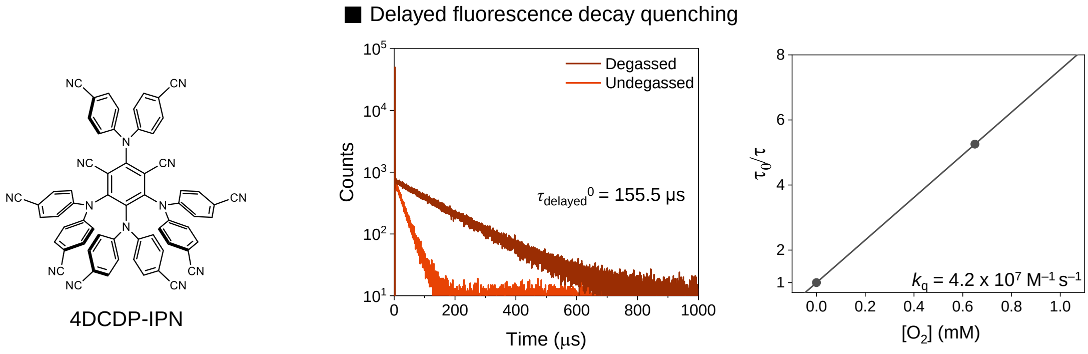

Supplementary Fig. 19 Stern-Volmer plots of 4DCDP-IPN with oxygen. The sample was fully saturated to air before the measurements. Oxygen concentration in DMF was taken to the literature (0.65 mM).[32]

Supplementary Note 4. ATRP with photoredox/copper dual catalysis for synthesis of PMMA

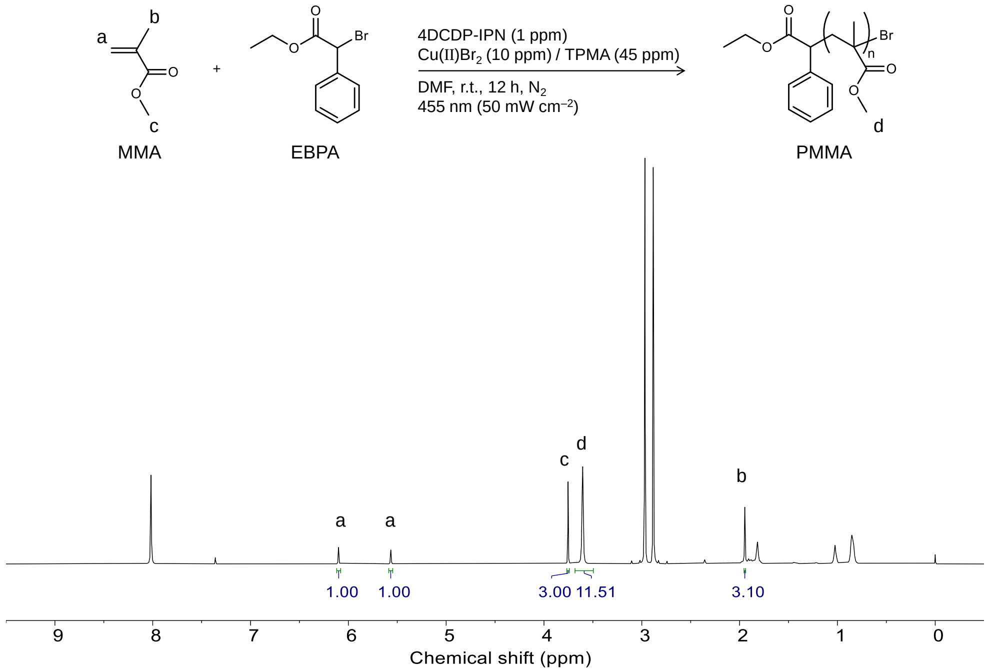

Supplementary Fig. 20 Representative ^{1}H-NMR spectrum of PMMA in $CDCl_3$. Conversion was determined by integral following relationship, $\alpha = I^{3.50–3.68} / (I^{3.74–3.77} + I^{3.50–3.68})$.

Supplementary Table 6 Control experiments in ATRP with photoredox/copper dual catalysis.

Entry	PC	PC loading (ppm)	$Cu(II)Br_2$ (ppm)	Ligand (ppm)	α (%)	$M_{n,theo}$ (g mol^{-1})[c]	$M_{n,exp}$ (g mol^{-1})[d]	I*[e]	Đ[f]
1	-	-	10	45	20	4,300	5,300	0.81	1.36
2	4DCDP-IPN	0.05	-	-	42	8,600	80,300	0.11	2.35
3[a]	4DCDP-IPN	0.05	10	45	No polymerization				
4[b]	4DCDP-IPN	0.05	10	45	54	-	168,200	-	3.54
5	4DCDP-IPN	0.05	10	10	No polymerization				
6	4DCDP-IPN	0.05	-	45	16	3,300	59,500	0.06	1.77
7	4DCDP-IPN	0.05	10	45	71	14,400	16,700	0.86	1.20

Polymerizations were carried out under the irradiation of 455 nm (50 mW cm^{-2}) for 24 h at r.t. Conversion was determined by ^{1}H NMR. [a]Reaction was conducted under the dark or [b]without the initiator. [c]$M_{n,theo} = [MMA]_0/[EBPA]_0 \times \text{conversion} \times M_{n,MMA} + M_{n,EBPA}$. [d]Determined by GPC using PMMA standards. [e]$I^* = M_{n,theo}/M_{n,exp}$. [f]$Đ = M_{w,exp}/M_{n,exp}$. Reaction condition: $[MMA]_0:[EBPA]_0:[4DCDP\text{-}IPN]_0:[Cu(II)Br_2]_0:[TPMA]_0$ = [200]:[1]:[0.00001]:[0.002]:[0.009], MMA/DMF = 1/1 (v/v).

Supplementary Table 7 Results of ATRP with photoredox/copper dual catalysis depending on the amount of ligand.

Entry	PC	PC loading (ppm)	Cu(II)Br$_2$ (ppm)	Ligand (ppm)	α (%)	$M_{n,theo}$ (g mol^{-1})^a	$M_{n,exp}$ (g mol^{-1})^b	I*^{c}	Đ^d
1	4DCDP-IPN	0.05	10	10	No polymerization				
2				20	53	10,900	11,600	0.94	1.22
3				45	57	11,700	13,100	0.89	1.21
4				100	64	13,100	14,900	0.88	1.20
5				200	69	14,000	15,200	0.92	1.20

Polymerizations were carried out under the irradiation of 455 nm (50 mW cm^{-2}) for 24 h at r.t. Conversion was determined by ^{1}H NMR. $^a M_{n,theo}$ = [MMA]$_0$/[EBPA]$_0$ × conversion × $M_{n,MMA}$ + $M_{n,EBPA}$. ^{b}Determined by GPC using PMMA standards. $^c I^* = M_{n,theo}/M_{n,exp}$. $^d Đ = M_{w,exp}/M_{n,exp}$. Reaction condition: [MMA]$_0$:[EBPA]$_0$:[4DCDP-IPN]$_0$:[Cu(II)Br$_2$]$_0$:[TPMA]$_0$ = [200]:[1]:[0.00001]:[0.002]:[x], MMA/DMF = 1/1 (v/v), x = 4 x 10^{-2}, 2 x 10^{-2}, 9 x 10^{-3}, 4 x 10^{-3} or 2 x 10^{-3} (200 ppm, 100 ppm, 45 ppm, 20 ppm and 10 ppm relative to the monomer, respectively).

Supplementary Table 8 Results of ATRP with photoredox/copper dual catalysis depending on the amount of Cu(II)Br$_2$.

Entry	PC	PC loading (ppm)	Cu(II)Br$_2$ (ppm)	α (%)	$M_{n,theo}$ (g mol^{-1})^a	$M_{n,exp}$ (g mol^{-1})^b	I*^{c}	Đ^d
1	4DCDP-IPN	1	100	83	16,800	16,400	1.02	1.13
2			50	94	19,100	16,600	1.15	1.26
3			10	89	18,100	20,300	0.89	1.35
4			5	89	18,100	15,300	1.18	1.32

Polymerizations were carried out under the irradiation of 455 nm (50 mW cm^{-2}) for 24 h at r.t. Conversion was determined by ^{1}H NMR. $^a M_{n,theo}$ = [MMA]$_0$/[EBPA]$_0$ × conversion × $M_{n,MMA}$ + $M_{n,EBPA}$. ^{b}Determined by GPC using PMMA standards. $^c I^* = M_{n,theo}/M_{n,exp}$. $^d Đ = M_{w,exp}/M_{n,exp}$. Reaction condition: [MMA]$_0$:[EBPA]$_0$:[4DCDP-IPN]$_0$:[Cu(II)Br$_2$]$_0$:[TPMA]$_0$ = [200]:[1]:[0.0002]:[x]:[4.5x], MMA/DMF = 1/1 (v/v), x = 2 x 10^{-2}, 1 x 10^{-1}, 2 x 10^{-3} or 1 x 10^{-3} (100 ppm, 50 ppm, 10 ppm and 5 ppm relative to the monomer, respectively).

Supplementary Table 9 PC screening in ATRP with photoredox/copper dual catalysis.

Entry	PC	PC loading (ppm)	$Cu(II)Br_2$ (ppm)	α (%)	$M_{n,theo}$ (g mol^{-1})[b]	$M_{n,exp}$ (g mol^{-1})[c]	I*[d]	Đ[e]
1[a]		50		53	10,800	13,300	0.81	1.11
2[a]	Eosin Y	25	100	42	8,600	10,100	0.84	1.11
3[a]		10		21	4,500	6,300	0.71	1.13
4[a]		50		95	19,200	22,000	0.87	1.24
5[a]		25		46	9,400	12,600	0.74	1.23
6[a]	Eosin Y	10	10	25	5,200	7,200	0.73	1.28
7[a]		5		20	4,200	4,500	0.93	1.30
8[a]		1		No polymerization				
7		1		66	13,400	15,900	0.84	1.22
8	*fac*-$Ir(ppy)_3$	0.1	10	37	7,600	9,600	0.79	1.27
9		0.05		30	6,200	8,300	0.75	1.29
10		1		57	11,700	14,100	0.83	1.22
11	Perylene	0.1	10	24	5,000	6,900	0.72	1.29
12		0.05		21	4,400	6,400	0.68	1.30
13[a]		10		91	18,500	18,700	0.99	1.23
14[a]	Rhodamine 6G	1	10	44	9,100	10,700	0.85	1.23
15[a]		0.1		No polymerization				

Polymerizations were carried out under the irradiation of 455 nm (50 mW cm^{-2}) for 24 h at r.t. Conversion was determined by ^{1}H NMR. [a]Polymerizations were conducted under the irradiation of 515 nm (50 mW cm^{-2}). [b]$M_{n,theo}$ = $[MMA]_0/[EBPA]_0$ × conversion × $M_{n,MMA}$ + $M_{n,EBPA}$. [c]Determined by GPC using PMMA standards. [d]I* = $M_{n,theo}/M_{n,exp}$. [e]Đ = $M_{w,exp}/M_{n,exp}$. Reaction condition: $[MMA]_0$:$[EBPA]_0$:$[4DCDP\text{-}IPN]_0$:$[Cu(II)Br_2]_0$:$[TPMA]_0$ = [200]:[1]:[x]:[y]:[0.009], MMA/DMF = 1/1 (v/v), x = 1 x 10^{-2}, 5 x 10^{-3}, 2 x 10^{-3}, 2 x 10^{-4}, 2 x 10^{-5} or 1 x 10^{-5}(50 ppm, 25 ppm, 10 ppm, 1 ppm, 0.1 ppm and 50 ppb relative to the monomer, respectively), y = 2 x 10^{-2} and 2 x 10^{-3} (100 ppm and 10 ppm relative to the monomer, respectively).

Supplementary Table 10 Cyanoarene-based PCs screening in ATRP with photoredox/copper dual catalysis.

Entry	PC	PC loading (ppm)	Cu(II)Br_2 (ppm)	α (%)	$M_{n,theo}$ (g mol^{-1})[a]	$M_{n,exp}$ (g mol^{-1})[b]	I*[c]	Đ[d]
1		1		61	12,500	14,000	0.89	1.22
2	4DMDP-IPN	0.1	10	20	4,300	5,000	0.86	1.40
3		0.05		9	1,900	2,300	0.84	1.52
4		1		93	18,900	22,000	0.86	1.34
5		0.5		76	15,400	16,700	0.93	1.18
7	4DP-IPN	0.1	10	70	14,200	18,800	0.75	1.17
8		0.05		49	10,000	12,200	0.82	1.18
9		0.01		32	6,700	9,100	0.74	1.44
10		1		75	15,200	15,400	0.99	1.34
11	4tCz-IPN	0.1	10	53	10,800	10,500	1.03	1.27
12		0.05		13	2,800	3,100	0.92	1.20
13		1		89	18,100	16,300	1.11	1.21
14		0.5		80	16,200	17,500	0.93	1.17
15	4Cz-IPN	0.1	10	68	13,800	14,500	0.95	1.17
16		0.05		48	9,900	12,800	0.77	1.20
17		0.01		41	8,500	7,400	1.14	1.29
18		1		86	17,400	17,300	1.01	1.24
19		0.5		89	18,000	17,400	1.03	1.22
20		0.1		69	14,100	15,400	0.91	1.19
21	4DCDP-IPN	0.05	10	68	13,800	16,300	0.85	1.17
22		0.025		40	8,300	9,500	0.87	1.26
23		0.01		43	8,900	10,900	0.82	1.23

Polymerizations were carried out under the irradiation of 455 nm (50 mW cm^{-2}) for 24 h at r.t. Conversion was determined by ^{1}H NMR. [a]$M_{n,theo}$ = $[MMA]_0/[EBPA]_0$ × conversion × $M_{n,MMA}$ + $M_{n,EBPA}$. [b]Determined by GPC using PMMA standards. [c]I* = $M_{n,theo}/M_{n,exp}$. [d]Đ = $M_{w,exp}/M_{n,exp}$. Reaction condition: $[MMA]_0:[EBPA]_0:[4DCDP\text{-}IPN]_0:[Cu(II)Br_2]_0:[TPMA]_0$ = [200]:[1]:[x]:[0.002]:[0.009], MMA/DMF = 1/1 (v/v), x = 2 x 10^{-4}, 2 x 10^{-5}, 1 x 10^{-5}, 5 x 10^{-6} or 2 x 10^{-6} (1 ppm, 0.1 ppm, 50 ppb, 25 ppb and 10 ppb relative to the monomer, respectively).

Supplementary Table 11 Reproducibility test in ATRP with photoredox/copper dual catalysis.

Entry	PC	PC loading (ppm)	Cu(II)Br$_2$ (ppm)	α (%)	$M_{n,theo}$ (g mol^{-1})[a]	$M_{n,exp}$ (g mol^{-1})[b]	I*[c]	Đ[d]
1-1	4DP-IPN	1	10	93	18,900	22,100	0.86	1.34
1-2				97	19,700	20,800	0.95	1.31
2-1	4DP-IPN	0.1	10	70	14,200	18,900	0.75	1.17
2-2				71	14,500	15,400	0.94	1.20
3-1	4DP-IPN	0.05	10	49	10,000	12,300	0.82	1.18
3-2				47	9,600	12,200	0.79	1.20
3-3				46	9,500	11,700	0.81	1.24
3-4				41	8,500	9,300	0.92	1.26
4-1	4Cz-IPN	1	10	89	18,100	16,300	1.11	1.21
4-2				96	19,500	20,800	0.94	1.28
5-1	4Cz-IPN	0.1	10	68	13,800	14,500	0.95	1.17
5-2				60	12,200	12,200	1.00	1.22
6-1	4Cz-IPN	0.05	10	48	9,900	12,800	0.77	1.20
6-2				55	11,800	11,100	1.06	1.32
6-3				46	9,500	9,200	1.04	1.28
7-1	4DCDP-IPN	1	10	86	17,400	17,300	1.01	1.24
7-2				89	18,100	20,300	0.89	1.35
8-1	4DCDP-IPN	0.1	10	69	14,060	15,400	0.91	1.19
8-2				85	17,200	17,900	0.96	1.21
9-1	4DCDP-IPN	0.05	10	68	13,800	16,300	0.85	1.17
9-2				71	14,400	16,700	0.86	1.20
9-3				61	12,400	14,100	0.87	1.17
9-4				69	14,100	16,200	0.87	1.17
9-5				63	12,800	14,000	0.91	1.17
9-6				71	14,500	16,100	0.90	1.20
9-7				57	11,700	13,100	0.89	1.21
10-1	4DCDP-IPN	0.025	10	40	8,300	9,500	0.87	1.26
10-2				16	3,400	4,000	0.85	1.36
10-3				60	12,300	10,100	1.22	1.20
10-4				9	2,100	2,600	0.82	1.33

Polymerizations were carried out under the irradiation of 455 nm (50 mW cm^{-2}) for 24 h at r.t. Conversion was determined by ^{1}H NMR. [a]$M_{n,theo}$ = $[MMA]_0/[EBPA]_0 \times$ conversion $\times M_{n,MMA} + M_{n,EBPA}$. [b]Determined by GPC using PMMA standards. [c]$I^* = M_{n,theo}/M_{n,exp}$. [d]$Đ = M_{w,exp}/M_{n,exp}$. Reaction condition: $[MMA]_0:[EBPA]_0:[PC]_0:[Cu(II)Br_2]_0:[TPMA]_0$ = [200]:[1]:[x]:[0.002]:[0.009], MMA/DMF = 1/1 (v/v). x = 2 x 10^{-4}, 2 x 10^{-5}, 1 x 10^{-5} or 5 x 10^{-6} (1 ppm, 0.1 ppm, 50 ppb and 25 ppb relative to the monomer, respectively).

Supplementary Table 12 Results of ATRP with photoredox/copper dual catalysis with PC at 1 ppm over 8 or 12 h.

Entry	PC	PC loading (ppm)	$Cu(II)Br_2$ (ppm)	Time (h)	α (%)	$M_{n,theo}$ (g mol^{-1})[a]	$M_{n,exp}$ (g mol^{-1})[b]	I*[c]	Đ[d]
1	4DCDP-IPN	1	10	8	73	14,800	14,900	1.00	1.28
2				12	79	16,100	16,800	0.96	1.30
3	4DP-IPN	1	10	8	64	13,100	13,200	0.99	1.25
4				12	74	15,000	16,100	0.93	1.24
5	4Cz-IPN	1	10	8	49	10,000	9,400	1.06	1.25
6				12	69	14,000	14,300	0.98	1.22

Polymerizations were carried out under the irradiation of 455 nm (50 mW cm^{-2}) for 8 or 12 h at r.t. Conversion was determined by ^{1}H NMR. [a]$M_{n,theo}$ = $[MMA]_0/[EBPA]_0$ × conversion × $M_{n,MMA}$ + $M_{n,EBPA}$. [b]Determined by GPC using PMMA standards. [c]I* = $M_{n,theo}/M_{n,exp}$. [d]Đ = $M_{w,exp}/M_{n,exp}$. Reaction condition: $[MMA]_0$:$[EBPA]_0$:$[4DCDP\text{-}IPN]_0$:$[Cu(II)Br_2]_0$:$[TPMA]_0$ = [200]:[1]:[0.0002]:[0.002]:[0.009], MMA/DMF = 1/1 (v/v).

Supplementary Table 13 Results of ATRP with photoredox/copper dual catalysis under undegassed condition.

Entry	PC	PC loading (ppm)	$Cu(II)Br_2$ (ppm)	α (%)	$M_{n,theo}$ (g mol^{-1})[a]	$M_{n,exp}$ (g mol^{-1})[b]	I*[c]	Đ[d]
1		1		91	18,400	21,300	0.86	1.30
2	4DP-IPN	0.1	10	69	14,000	17,100	0.82	1.31
3		0.05		56	11,400	14,700	0.78	1.22
4		1		91	18,500	21,500	0.86	1.28
5	4Cz-IPN	0.1	10	74	15,100	18,300	0.82	1.21
6		0.05		66	13,500	16,700	0.81	1.22
7		1		96	19,400	25,000	0.78	1.34
8	4DCDP-IPN	0.1	10	78	15,900	21,700	0.73	1.18
9		0.05		66	13,500	19,200	0.71	1.22

Polymerizations were carried out undegassed condition under the irradiation of 455 nm (50 mW cm^{-2}) for 24 h at r.t. 2 mL of the reaction mixture was added to a 4 mL sealed vial. Conversion was determined by ^{1}H NMR. [a]$M_{n,theo}$ = $[MMA]_0/[EBPA]_0$ × conversion × $M_{n,MMA}$ + $M_{n,EBPA}$. [b]Determined by GPC using PMMA standards. [c]I* = $M_{n,theo}/M_{n,exp}$. [d]Đ = $M_{w,exp}/M_{n,exp}$. Reaction condition: $[MMA]_0$:$[EBPA]_0$:$[PC]_0$:$[Cu(II)Br_2]_0$:$[TPMA]_0$ = [200]:[1]:[x]:[0.002]:[0.009], MMA/DMF = 1/1 (v/v), x = 2 x 10^{-4}, 2 x 10^{-5} or 1 x 10^{-5} (1 ppm, 0.1 ppm and 50 ppb relative to the monomer, respectively).

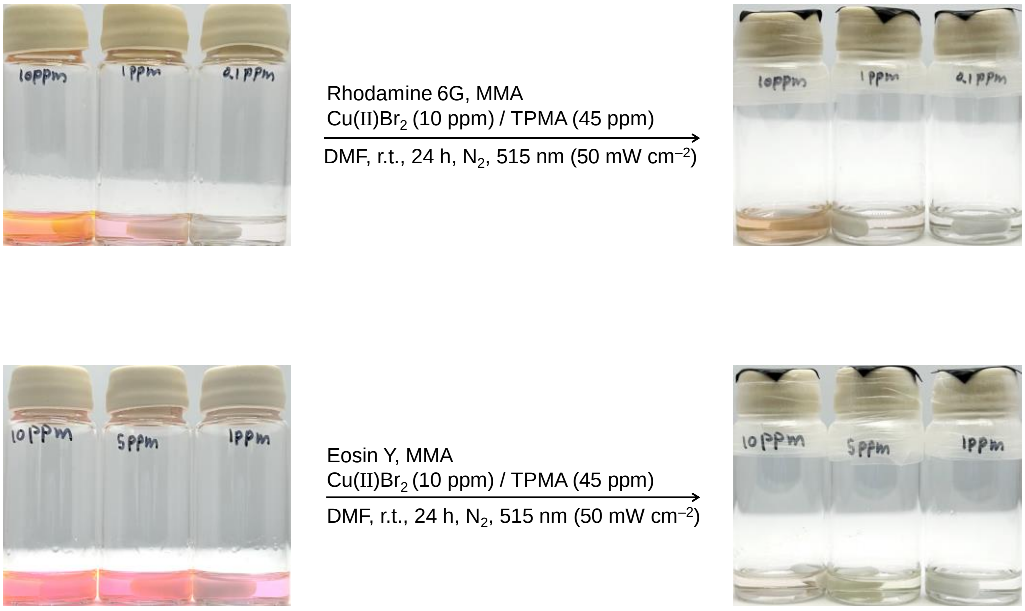

Supplementary Fig. 21 PC degradation during the polymerization. Images taken before the polymerization reaction (left) and after the polymerization reaction (right) are given.

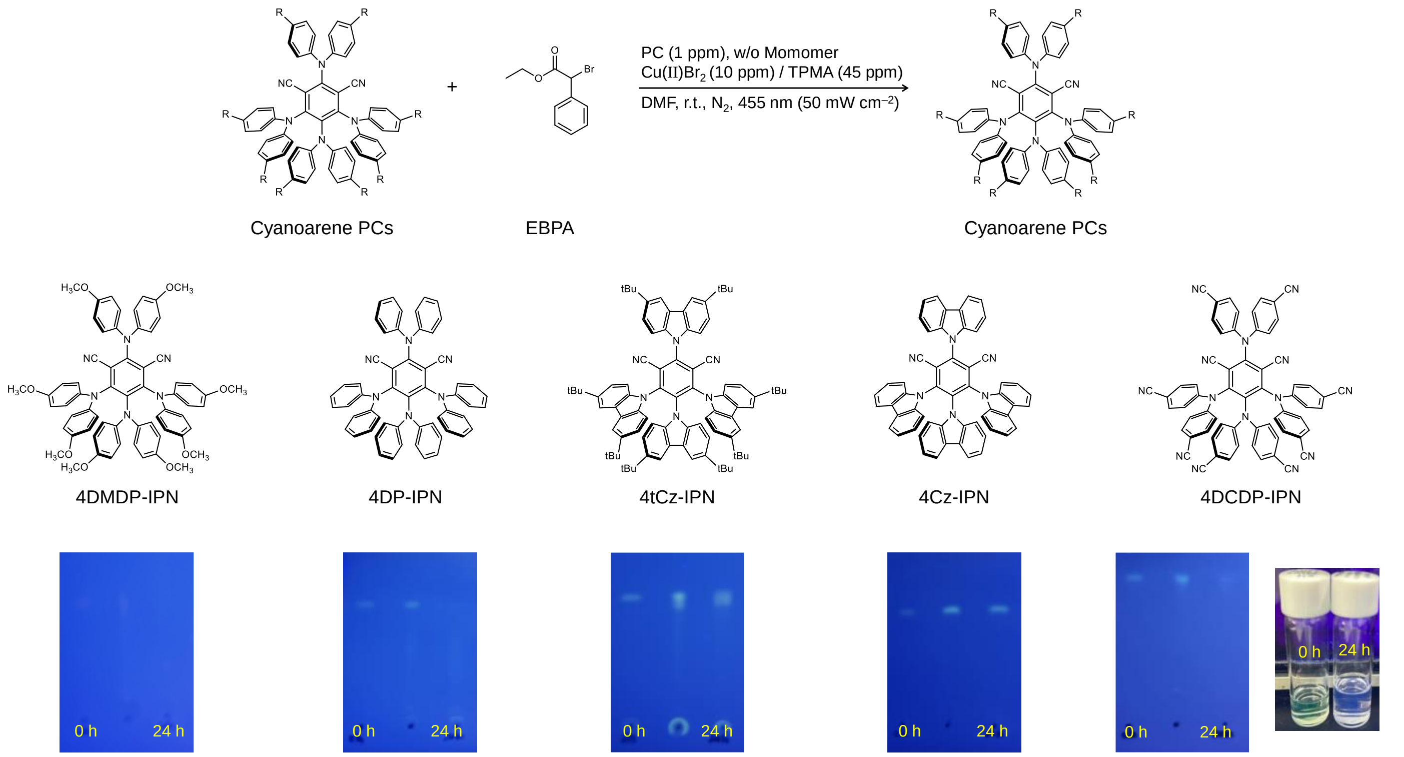

Supplementary Fig. 22 Thin layer chromatography (TLC) screening without MMA was conducted before and after irradiation of 455 nm (50 mW cm^{-2}). In the case of 4DMDP-IPN, emitting spots are invisible in images because its PLQY is very low.

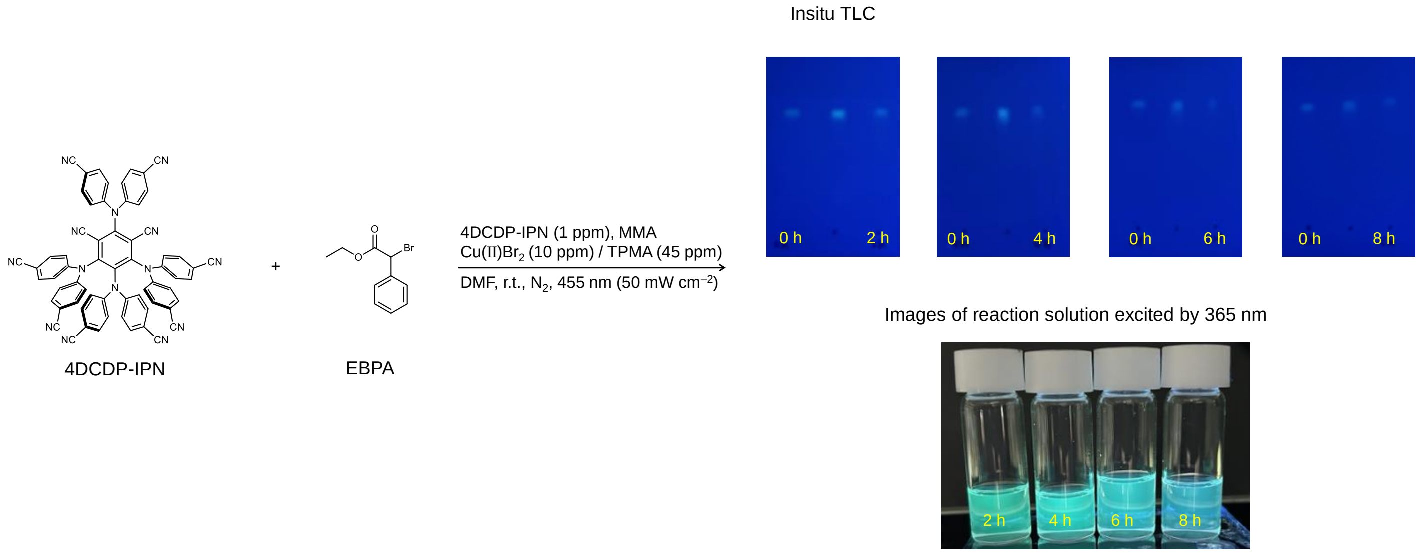

Supplementary Fig. 23 Monitoring in-situ TLC of polymerization sample conducted with 4DCDP-IPN at 1 ppm under irradiation of 455 nm (50 mW cm^{-2}). In-situ TLC was monitored every 2 h (top). To visualize clearly, after in-situ polymerization samples are diluted in DCM, PL emission was recorded under 365 nm excitation (bottom).

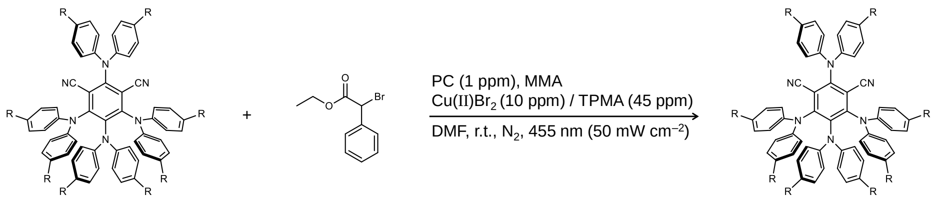

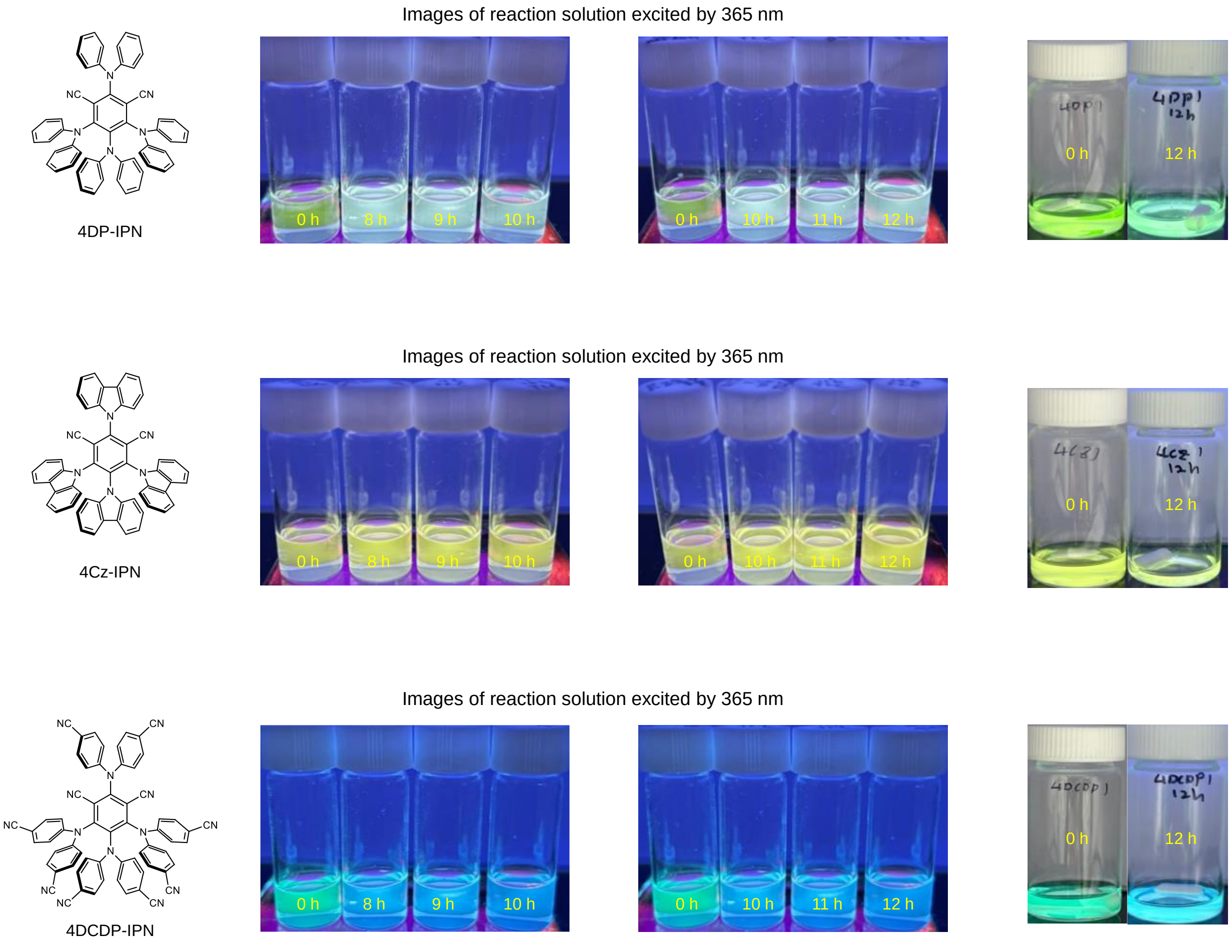

Supplementary Fig. 24 Monitoring PL emission of in-situ samples conducted with 4DP-IPN, 4Cz-IPN and 4DCDP-IPN at 1 ppm under irradiation of 455 nm (50 mW cm^{-2}). To visualize clearly, after in-situ polymerization samples are diluted in DCM, PL emission was recorded under 365 nm excitation.

Supplementary Table 14 Kinetic experiments in ATRP with photoredox/copper dual catalysis with 4DCDP-IPN at 50 ppb.

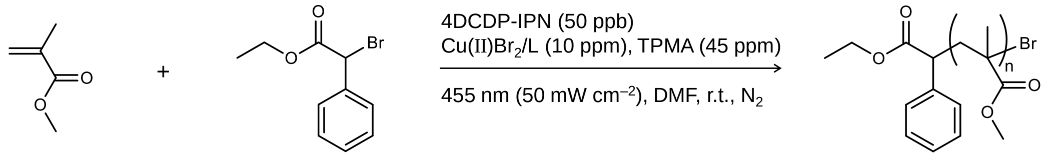

MMA EBPA PMMA

Entry	PC	PC loading (ppm)	Cu(II)Br$_2$ (ppm)	Time (h)	α (%)	$M_{n,theo}$ (g mol^{-1})[a]	$M_{n,exp}$ (g mol^{-1})[b]	I*[c]	Đ[d]
1				4	14	2,900	5,400	0.55	1.29
2				8	34	7,100	9,200	0.77	1.28
3	4DCDP-IPN	0.05	10	12	48	9,900	11,800	0.83	1.25
4				16	60	12,200	13,700	0.89	1.23
5				20	67	13,700	15,100	0.91	1.22
6				24	71	14,500	16,100	0.90	1.20

Polymerizations were carried out under the irradiation of 455 nm (50 mW cm^{-2}) at r.t. Conversion was determined by ^{1}H NMR. [a]$M_{n,theo}$ = $[MMA]_0/[EBPA]_0$ × conversion × $M_{n,MMA}$ + $M_{n,EBPA}$. [b]Determined by GPC using PMMA standards. [c]I* = $M_{n,theo}/M_{n,exp}$. [d]Đ = $M_{w,exp}/M_{n,exp}$. Reaction condition: $[MMA]_0:[EBPA]_0:[4DCDP\text{-}IPN]_0:[Cu(II)Br_2]_0:[TPMA]_0$ = [200]:[1]:[0.00001]:[0.002]:[0.009], MMA/DMF = 1/1 (v/v).

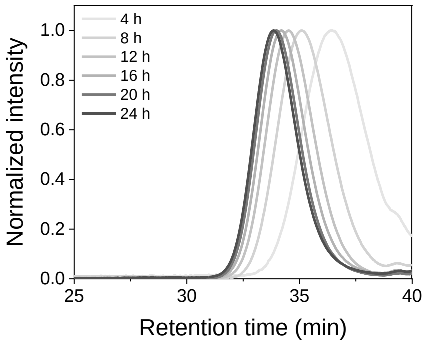

Supplementary Fig. 25 GPC traces of the synthesized polymers in Supplementary Table 14.

Supplementary Table 15 Initial kinetic experiments in ATRP with photoredox/copper dual catalysis with PCs at 1 ppm.

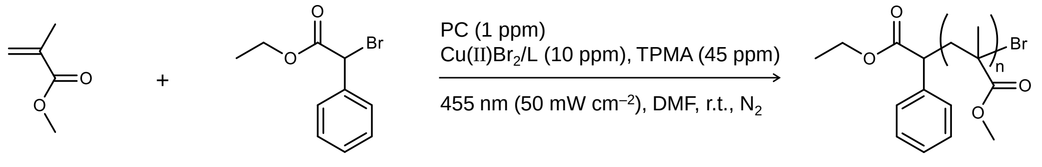

MMA EBPA PMMA

Entry	PC	PC loading (ppm)	Cu(II)Br2 (ppm)	Time (h)	α (%)	$M_{n,theo}$ (g mol^{-1})[a]	$M_{n,exp}$ (g mol^{-1})[b]	I*[c]	Đ[d]
1				1	9	2,100	-	-	-
2				2	21	4,500	5,400	0.83	1.31
3	4DP-IPN	1	10	4	40	8,300	8,900	0.93	1.27
4				6	53	10,900	11,600	0.94	1.24
5				8	64	13,100	13,200	0.99	1.25
6				1	4	900	-	-	-
7				2	10	2,200	3,200	0.68	1.26
8	4Cz-IPN	1	10	4	26	5,400	5,600	0.97	1.30
9				6	39	8,000	7,500	1.06	1.27
10				8	49	10,000	9,400	1.06	1.25
11				1	8	1,800	-	-	-
12				2	21	4,500	5,900	0.76	1.32
13	4DCDP-IPN	1	10	4	44	9,100	10,300	0.88	1.26
14				6	61	12,400	13,100	0.95	1.27
15				8	73	14,800	14,900	1.00	1.28

Polymerizations were carried out under the irradiation of 455 nm (50 mW cm^{-2}) for 24 h at r.t. Conversion was determined by ^{1}H NMR. [a]$M_{n,theo}$ = $[MMA]_0/[EBPA]_0$ × conversion × $M_{n,MMA}$ + $M_{n,EBPA}$. [b]Determined by GPC using PMMA standards. [c]I* = $M_{n,theo}/M_{n,exp}$. [d]Đ = $M_{w,exp}/M_{n,exp}$. Reaction condition: $[MMA]_0:[EBPA]_0:[PC]_0:[Cu(II)Br_2]_0:[TPMA]_0$ = [200]:[1]:[0.0002]:[0.002]:[0.009], MMA/DMF = 1/1 (v/v).

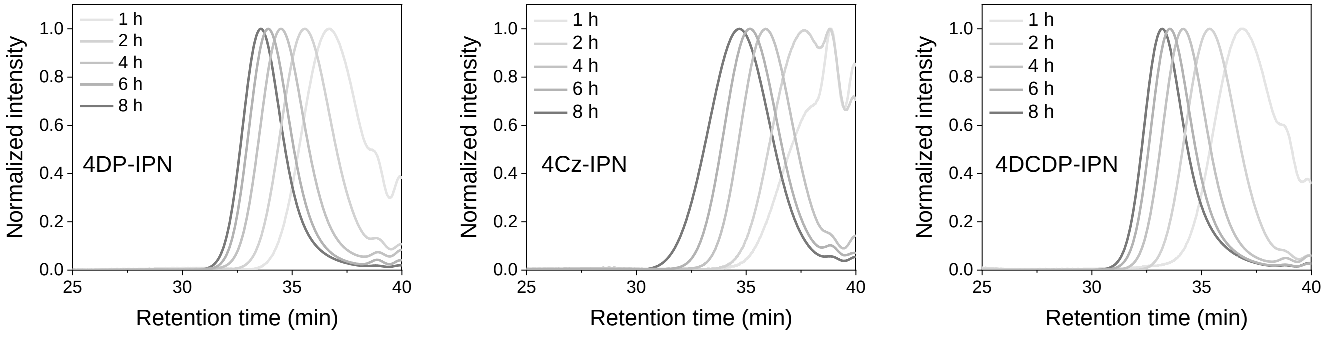

Supplementary Fig. 26 GPC traces of the synthesized polymers in Supplementary Table 15.

Supplementary Table 16 Initial kinetic experiments in ATRP with photoredox/copper dual catalysis with PCs at 50 ppb.

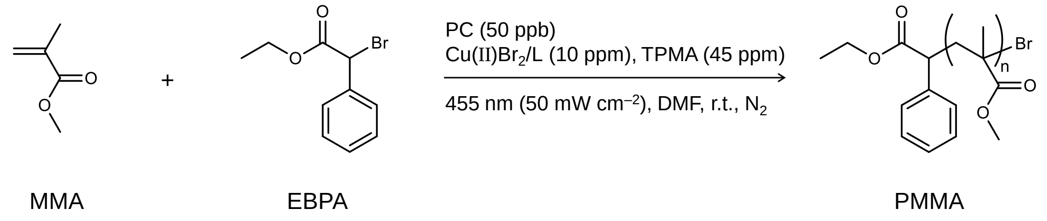

Entry	PC	PC loading (ppm)	$Cu(II)Br_2$ (ppm)	Time (h)	α (%)	$M_{n,theo}$ (g mol^{-1})[a]	$M_{n,exp}$ (g mol^{-1})[b]	I*[c]	Đ[d]
1	4DP-IPN	0.05	10	1	3	800	-	-	-
2				2	5	1,300	-	-	-
3				4	10	2,200	-	-	-
4				6	15	3,100	4,000	0.78	1.31
5				8	16	3,500	4,200	0.83	1.29
6	4Cz-IPN	0.05	10	1	-	-	-	-	-
7				2	1	-	-	-	-
8				4	3	800	-	-	-
9				6	5	1,300	-	-	-
10				8	7	1,600	-	-	-
11	4DCDP-IPN	0.05	10	1	3	800	-	-	-
12				2	5	1,300	-	-	-
13				4	13	2,700	3,600	0.76	1.28
14				6	22	4,500	4,800	0.95	1.30
15				8	27	5,700	5,700	1.00	1.31

Polymerizations were carried out under the irradiation of 455 nm (50 mW cm^{-2}) at r.t. Conversion was determined by ^{1}H NMR. [a]$M_{n,theo}$ = $[MMA]_0/[EBPA]_0$ × conversion × $M_{n,MMA}$ + $M_{n,EBPA}$. [b]Determined by GPC using PMMA standards. [c]I* = $M_{n,theo}/M_{n,exp}$. [d]Đ = $M_{w,exp}/M_{n,exp}$. Reaction condition: $[MMA]_0$:$[EBPA]_0$:$[PC]_0$:$[Cu(II)Br_2]_0$:$[TPMA]_0$ = [200]:[1]:[0.00001]:[0.002]:[0.009], MMA/DMF = 1/1 (v/v).

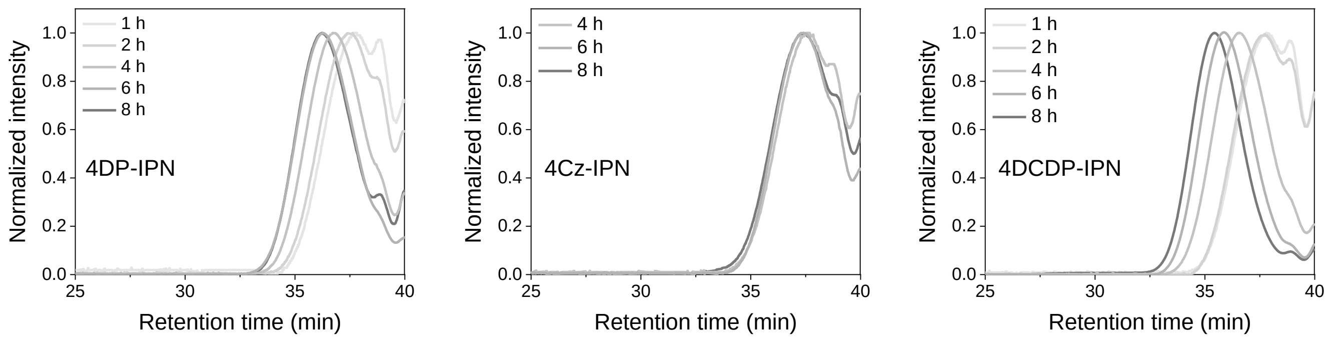

Supplementary Fig. 27 GPC traces of the synthesized polymers in Supplementary Table 16.

Supplementary Table 17 Kinetic experiments in ATRP with photoredox/copper dual catalysis with 4DCDP-IPN at 1 ppm or 50 ppb under undegassed conditions over 0–8 h.

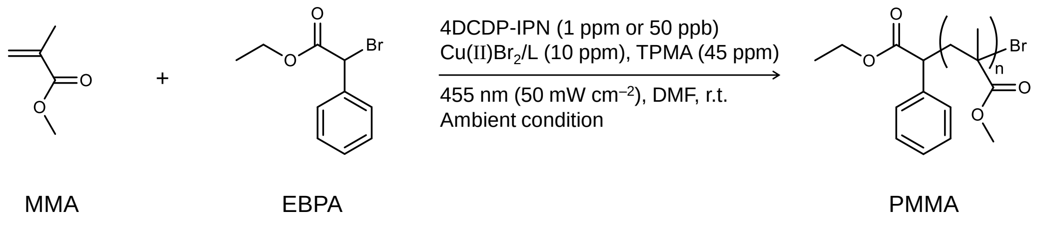

Entry	PC	PC loading (ppm)	Cu(II)Br₂ (ppm)	Time (h)	α (%)	$M_{n,theo}$ (g mol^{-1})[a]	$M_{n,exp}$ (g mol^{-1})[b]	I*[c]	Đ[d]
1				1	9	2,100	3,800	0.56	1.39
2				2	40	8,200	8,700	0.94	1.33
3	4DCDP-IPN	1	10	4	53	10,900	13,200	0.83	1.33
4				6	66	13,400	15,700	0.85	1.33
5				8	73	16,800	16,900	0.99	1.34
1				1	0	-	-	-	-
2				2	3	800	-	-	-
3	4DCDP-IPN	0.05	10	4	14	3,000	5,100	0.60	1.35
4				6	27	5,700	7,200	0.79	1.37
5				8	35	7,300	8,900	0.81	1.33

Polymerizations were carried out with undegassed condition under the irradiation of 455 nm (50 mW cm^{-2}) at r.t. Conversion was determined by ^{1}H NMR. [a]$M_{n,theo}$ = $[MMA]_0/[EBPA]_0$ × conversion × $M_{n,MMA}$ + $M_{n,EBPA}$. [b]Determined by GPC using PMMA standards. [c]I* = $M_{n,theo}/M_{n,exp}$. [d]Đ = $M_{w,exp}/M_{n,exp}$. Reaction condition: $[MMA]_0:[EBPA]_0:[PC]_0:[Cu(II)Br_2]_0:[TPMA]_0$ = [200]:[1]:[x]:[0.002]:[0.009], MMA/DMF = 1/1 (v/v). x = 2 x 10^{-4}, 1 x 10^{-5} (1 ppm and 50 ppb relative to the monomer, respectively).

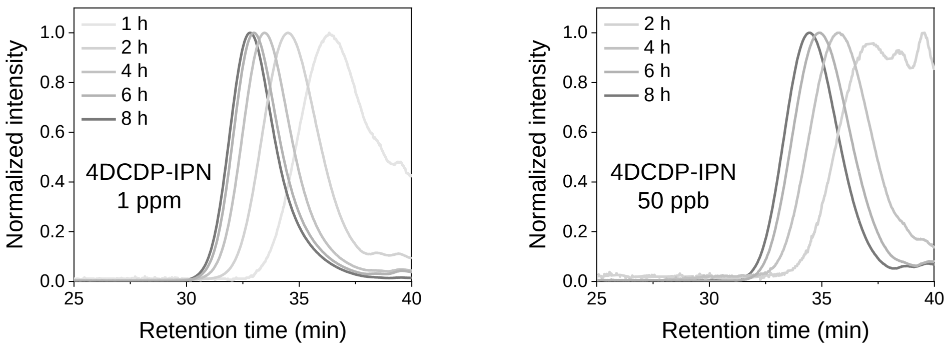

Supplementary Fig. 28 GPC traces of the synthesized polymers in Supplementary Table 17.

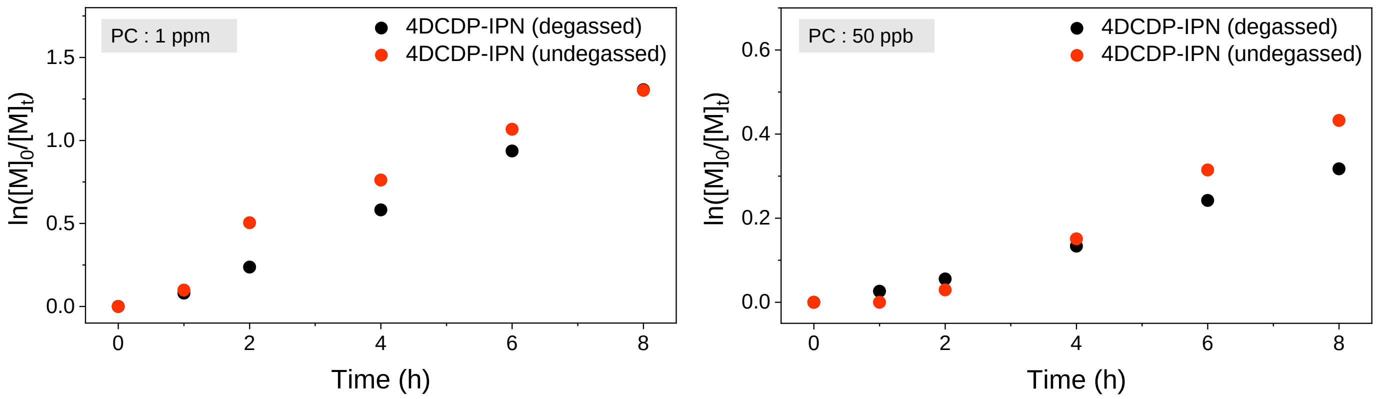

Supplementary Fig. 29 Comparison of kinetics under undegassed and degassed conditions with 4DCDP-IPN at 1 ppm or 50 ppb.

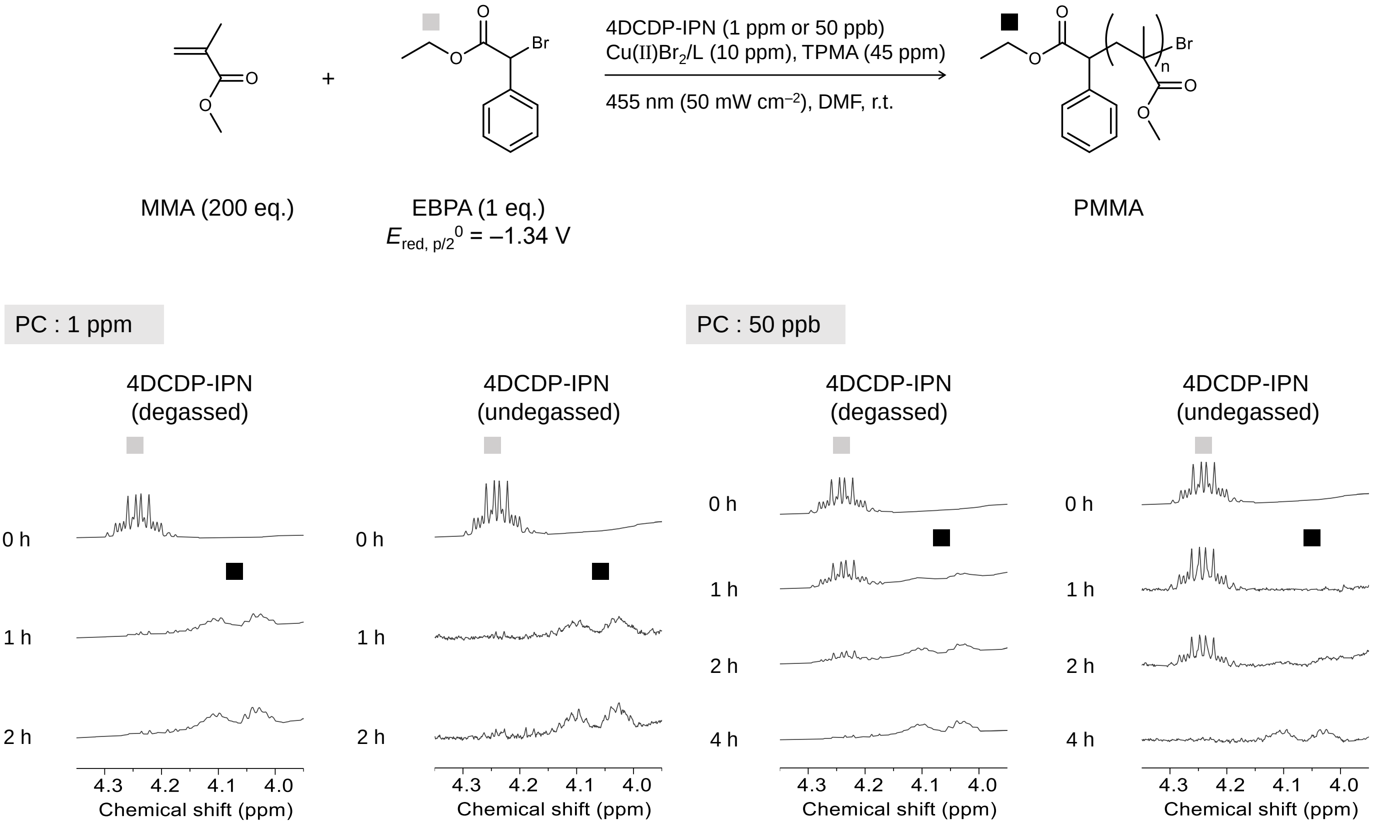

Supplementary Fig. 30 Conversion of EBPA under undegassed and degassed conditions with 4DCDP-IPN at 1 ppm and 50 ppb. The conversions of EBPA were monitored through in-situ ^{1}H NMR measurements.

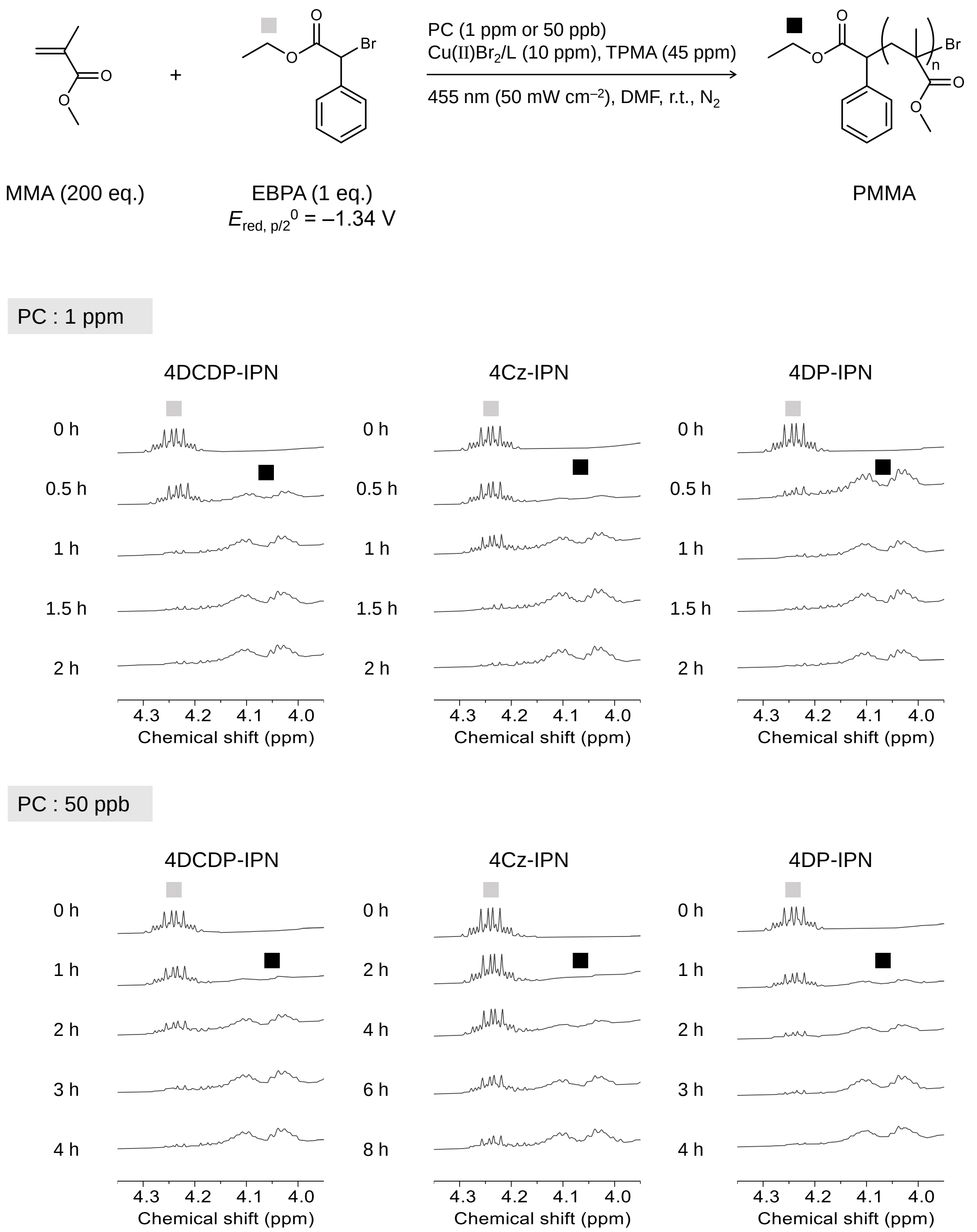

Supplementary Fig. 31 Conversion of EBPA with PCs at 1 ppm and 50 ppb. The conversions of EBPA were monitored through in-situ ^{1}H NMR measurements.

Supplementary Table 18 Methacrylate-based monomer scopes in ATRP with photoredox/copper dual catalysis.

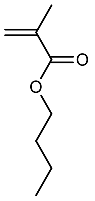

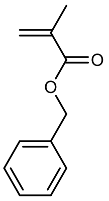

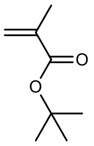

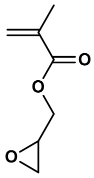

Butyl methacrylate | Benzyl methacrylate | tert-Butyl methacrylate | Glycidyl methacrylate

Entry	Monomer	PC loading (ppm)	Cu(II)Br$_2$ (ppm)	α (%)	$M_{n,theo}$ (g mol^{-1})^a	$M_{n,exp}$ (g mol^{-1})^b	I^{*c}	Đ^d
1		1		88	25,200	30,300	0.83	1.25
2	Butyl methacrylate	0.1	10	72	20,800	26,500	0.78	1.22
3		0.05		62	17,800	22,800	0.78	1.24
4		1		87	30,900	33,000	0.93	1.33
5	Benzyl methacrylate	0.1	10	82	29,100	31,700	0.92	1.28
6		0.05		63	22,500	23,300	0.97	1.34
7		1		59	17,100	23,800	0.72	1.29
8	tert-Butyl methacrylate	0.1	10	26	7,500	10,700	0.70	1.28
9		0.05		28	8,100	11,500	0.70	1.26
10		1		100	28,700	28,800	1.00	1.35
11	Glycidyl methacrylate	0.1	10	99	28,400	31,900	0.89	1.74
12		0.05		97	27,900	36,100	0.77	2.43

Polymerizations were carried out under the irradiation of 455 nm (50 mW cm^{-2}) for 24 h at r.t. Conversion was determined by ^{1}H NMR. $^a M_{n,theo}$ = $[MMA]_0/[EBPA]_0 \times$ conversion $\times M_{n,MMA} + M_{n,EBPA}$. ^{b}Determined by GPC using PMMA standards. $^c I^* = M_{n,theo}/M_{n,exp}$. $^d Đ = M_{w,exp}/M_{n,exp}$. Reaction condition: $[MMA]_0$:$[EBPA]_0$:$[4DCDP\text{-}IPN]_0$:$[Cu(II)Br_2]_0$:$[TPMA]_0$ = [200]:[1]:[x]:[0.002]:[0.009], MMA/DMF = 1/1 (v/v), x = 2 x 10^{-4} , 2 x 10^{-5} or 1 x 10^{-5} (1 ppm, 0.1 ppm and 50 ppb relative to the monomer, respectively).

Supplementary Table 19 Results of the polystyrene polymerizations in ATRP with photoredox/copper dual catalysis.

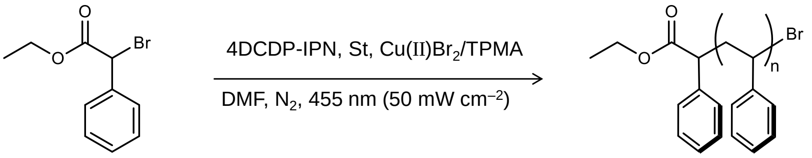

Entry	PC	PC loading (ppm)	Cu(II)Br2 (ppm)	Time (h)	α (%)	$M_{n,theo}$ (g mol^{-1})[b]	$M_{n,exp}$ (g mol^{-1})[c]	I*[d]	Đ[e]
1	4DCDP-IPN	10	100	24	18	4,100	4,800	0.85	1.12
2[a]	4DCDP-IPN	10	100	24	32	6,900	8,200	0.84	1.20
3	4DCDP-IPN	10	10	24	45	9,300	12,500	0.74	1.62
4	4DCDP-IPN	5	10	24	40	8,200	12,600	0.65	1.81
5	4DCDP-IPN	1	10	24	17	3,800	4,200	0.90	1.21
6				48	31	6,600	6,400	1.03	1.23
7[a]	4DCDP-IPN	1	10	24	32	6,900	6,600	1.05	1.36

Polymerizations were carried out under the irradiation of 455 nm (50 mW cm^{-2}) for 24 h at r.t. following the general procedure with condition, $[St]_0$:$[EBPA]_0$:$[PC]_0$:$[Cu(II)Br_2]_0$:$[TPMA]_0$ = [200]:[1]:[x]:[y]:[4.5y], MMA/DMF = 1/1 (v/v) where x = 2 x 10^{-3}, 1 x 10^{-3} or 2 x 10^{-4} (for 10 ppm, 5 ppm, and 1 ppm relative to the monomer, respectively), y = 2 x 10^{-2} or 2 x 10^{-3} (for 100 ppm and 10 ppm relative to the monomer, respectively). Conversion was determined by ^{1}H NMR. [a]Reaction was performed at temperature of 35 °C. [b]$M_{n,theo}$ was determined by following relationship, $M_{n,theo} = [St]_0/[EBPA]_0 \times \text{conversion} \times M_{n,St} + M_{n,EBPA}$. [c]$M_{n,exp}$ was measured by GPC equipped with refractive index detector calibrated with PMMA standards. [d]Initiator efficiency (I*) was determined by I* = $M_{n,theo}$ / $M_{n,exp}$. [e]Dispersity (Đ) was determined by Đ = $M_{w,exp}$ / $M_{n,exp}$.

Supplementary Table 20 Results of the poly(methyl acrylate) polymerizations in ATRP with photoredox/copper dual catalysis.

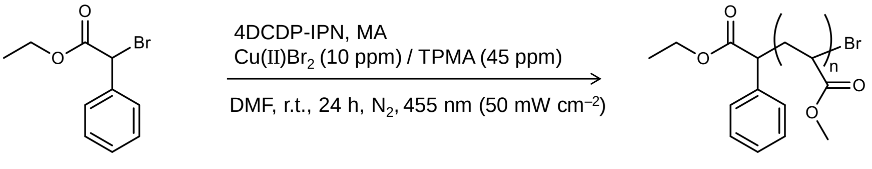

Entry	PC	PC loading (ppm)	Cu(II)Br2 (ppm)	α (%)	$M_{n,theo}$ (g mol^{-1})[a]	$M_{n,exp}$ (g mol^{-1})[b]	I*[c]	Đ[d]
1	4DCDP-IPN	1	10	83	14,600	14,200	1.03	1.47
2	4DCDP-IPN	0.1	10	17	3,200	3,900	0.82	1.26
3	4DCDP-IPN	0.05	10	13	2,400	4,900	0.49	1.35

Polymerizations were carried out under the irradiation of 455 nm (50 mW cm^{-2}) for 24 h at r.t. following the general procedure with condition, $[MA]_0$:$[EBPA]_0$:$[PC]_0$:$[Cu(II)Br_2]_0$:$[TPMA]_0$ = [200]:[1]:[x]:[0.002]:[0.009], MA/DMF = 1/1 (v/v) where x = 2 x 10^{-4}, 2 x 10^{-5} or 1 x 10^{-5} (for 1 ppm, 0.1 ppm and 50 ppb relative to the monomer, respectively). Conversion was determined by ^{1}H NMR. [a]$M_{n,theo}$ was determined by following relationship, $M_{n,theo} = [MA]_0/[EBPA]_0 \times \text{conversion} \times M_{n,MA} + M_{n,EBPA}$. [b]$M_{n,exp}$ was measured by GPC equipped with refractive index detector calibrated with PMMA standards. [c]Initiator efficiency (I*) was determined by I* = $M_{n,theo}/M_{n,exp}$. [d]Dispersity (Đ) was determined by Đ = $M_{w,exp}/M_{n,exp}$.

Supplementary Table 21 Results of polymerizations with various solvents in ATRP with photoredox/copper dual catalysis.

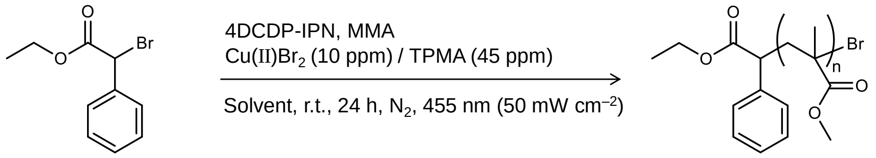

Entry	PC	PC loading (ppm)	Cu(II)Br2 (ppm)	Solvent	α (%)	$M_{n,theo}$ (g mol^{-1})[b]	$M_{n,exp}$ (g mol^{-1})[c]	I*[d]	Đ[e]
1		1			89	18,100	20,300	0.89	1.35
2	4DCDP-IPN	0.1	10	DMF	85	17,200	17,900	0.96	1.21
3		0.05			71	14,400	16,700	0.86	1.20
4		1			89	18,100	17,900	1.01	1.24
5	4DCDP-IPN	0.1	10	ACN	51	10,400	10,400	1.00	1.16
6		0.05			36	7,500	7,900	0.95	1.16
7[a]		1			70	14,300	12,300	1.16	1.19
8[a]	4DCDP-IPN	0.1	10	DCM	28	5,800	4,700	1.23	1.20
9[a]		0.05			20	4,300	3,800	1.13	1.19

Polymerizations were carried out under the irradiation of 455 nm (50 mW cm^{-2}) for 24 h at r.t. following the general procedure with condition, $[MMA]_0:[EBPA]_0:[4DCDP\text{-}IPN]_0:[Cu(II)Br_2]_0:[TPMA]_0$ = [200]:[1]:[x]:[0.002]:[0.009], MMA/Solvent = 1/1 (v/v) where x = 2 x 10^{-4}, 2 x 10^{-5} or 1 x 10^{-5} (for 1 ppm, 0.1 ppm and 50 ppb relative to the monomer, respectively). Monomer conversion (α) was determined by integrals of ^1H NMR peaks. [a]Reaction was conducted in a glove box. [b]$M_{n,theo}$ was determined by following relationship, $M_{n,theo} = [MMA]_0/[EBPA]_0 \times \alpha \times M_{n,MMA} + M_{n,EBPA}$. [c]$M_{n,exp}$ was measured by GPC equipped with refractive index detector calibrated with PMMA standards. [d]Initiator efficiency (I*) was determined by $I^* = M_{n,theo}/M_{n,exp}$. [e]Dispersity (Đ) was determined by $Đ = M_{w,exp}/M_{n,exp}$.

Supplementary Table 22 Synthesis of macroinitiators and block copolymers in ATRP with photoredox/copper dual catalysis.

Entry	PC	PC loading (ppm)	Cu(II)Br_2 (ppm)	Time (h)	α (%)	$M_{n,theo}$ (g mol^{-1})[a]	$M_{n,exp}$ (g mol^{-1})[b]	I*[c]	Đ[d]
In-situ	4DCDP-IPN	0.05	10	24	54	5,900	6,100	0.97	1.28
Isolation					-	-	7,500	-	1.22
PMMA-*b*-PMMA	4DCDP-IPN	0.05	10	24	-	-	12,600	-	1.25
PMMA-*b*-PBMA	4DCDP-IPN	0.05	10	24	-	-	16,100	-	1.28
PMMA-*b*-BzMA	4DCDP-IPN	0.05	10	24	-	-	14,500	-	1.37
In-situ	4DCDP-IPN	1	10	4	41	4,300	5,300	0.82	1.29
Isolation					-	-	6700	-	1.22
PMMA-*b*-PS	4DCDP-IPN	1	10	24	-	-	8,800	-	1.17

Polymerizations were carried out under the irradiation of 455 nm (50 mW cm^{-2}) at r.t. Monomer conversion (α) was determined by integrals of ^1H NMR peaks. [a]$M_{n,theo}$ = $[MMA]_0/[EBPA]_0$ × conversion × $M_{n,MMA}$ + $M_{n,EBPA}$. [c]Determined by GPC using PMMA standards. [d]I* = $M_{n,theo}/M_{n,exp}$. [e]Đ = $M_{w,exp}$ / $M_{n,exp}$. Reaction condition: $[MMA]_0$:$[EBPA]_0$:[4DCDP-IPN]:$[Cu(II)Br_2]_0$:[TPMA] = [100]:[1]:[x]:[0.001]:[0.0045], MMA/DMF = 1/1 (v/v), x = 1 x 10^{-4} or 5 x 10^{-6} (1 ppm and 50 ppb relative to the monomer). Block copolymer reaction condition: $[M]_0$:$[PMMA\text{-}Br]_0$:$[4DCDP\text{-}IPN]_0$:$[Cu(II)Br_2]_0$:$[TPMA]_0$ = [200]:[1]:[x]:[0.002]:[0.009] in anhydrous DMF (2 mL, sum of weight of (monomer + macroinitiator) / volume of DMF = 1/4 (w/v)), x = 2 x 10^{-4}, 1 x 10^{-5} (1 ppm and 50 ppb relative to the monomer).

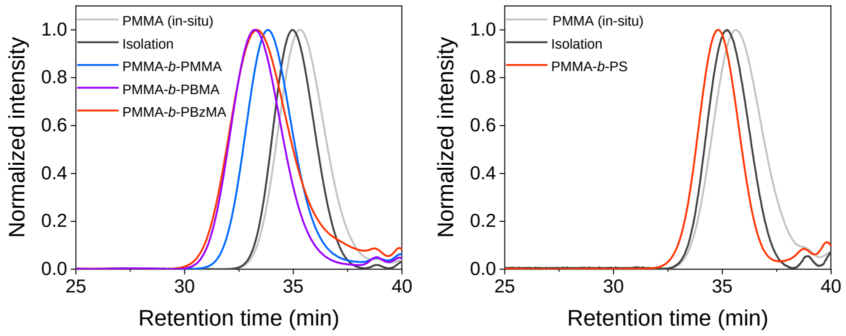

Supplementary Fig. 32 GPC traces of the synthesized polymers in Supplementary Table 22. Macroinitiator and block copolymer were synthesized with 4DCDP-IPN at 50 ppb (left) and 1 ppm (right).

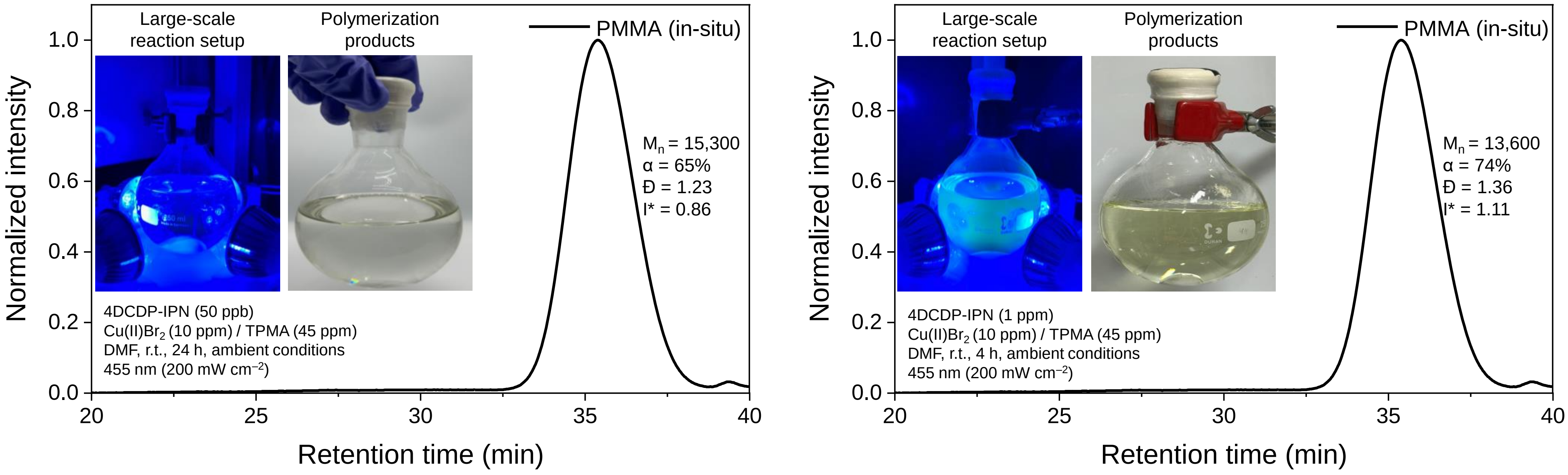

Supplementary Fig. 33 GPC traces of synthesized polymers in large-scale polymerization with 4DCDP-IPN at 50 ppb (left) and 1 ppm (right). Images of large-scale reaction setups and products are also given (inset).

Supplementary Note 5. Coordinates of molecular structures obtained by DFT calculation

The geometries optimizations of each species were obtained DFT calculations were performed with the B3LYP functional and 6–311++G* basis set ACN solution employing the PCM solvation model. as all implemented in the Gaussian16 program package (Revision C.01 x86_64 AVX-enabled Binary Version). In all geometry optimization calculations, the frequency calculations were performed both to verify that the geometries were true minima and to obtain thermochemistry-correction values.

■ 4,4'-dimethoxydiphenyl amine (neutral)

Electronic energy = -747.88266 (hartree)

Electronic energy + zero-point energy = -747.62109 (hartree)

Electronic energy + thermal energy correction = -747.60532 (hartree)

Electronic energy + thermal enthalpy correction = -747.60438 (hartree)

Electronic energy + thermal free energy correction = -747.66647 (hartree)

N	0.01585	-1.48797	-0.18856
H	0.06867	-2.44921	0.11546
C	-1.2707	-0.924	-0.10449
C	-1.51461	0.41965	0.19425
C	-2.38242	-1.7611	-0.31949
C	-2.81909	0.92123	0.26282
H	-0.69059	1.09437	0.3901
C	-3.67635	-1.27229	-0.23378
H	-2.22364	-2.80929	-0.55579
C	-3.91027	0.07933	0.05095
H	-2.95712	1.96983	0.4948
H	-4.52289	-1.93096	-0.39734
C	1.24173	-0.79734	-0.15022
C	1.47627	0.3453	-0.93494
C	2.29841	-1.29027	0.6218
C	2.71379	0.97215	-0.92505
H	0.69042	0.73578	-1.57109
C	3.55466	-0.67864	0.61656
H	2.1454	-2.17131	1.23819
C	3.767	0.4673	-0.15165
H	2.88519	1.85385	-1.53364

H	4.34334	-1.10235	1.2253
O	-5.22503	0.47032	0.09764
C	-5.51086	1.83263	0.40058
H	-5.08396	2.50523	-0.34957
H	-6.5954	1.92089	0.388
H	-5.13643	2.1068	1.39151
C	4.66562	2.59821	0.24164
H	4.12523	3.11658	-0.52267
H	4.0832	2.57796	1.13901
H	5.591	3.10193	0.42832
O	4.94977	1.15631	-0.21856

■ diphenyl amine (neutral)

Electronic energy = -518.77505 (hartree)

Electronic energy + zero-point energy = -518.57791 (hartree)

Electronic energy + thermal energy correction = -518.56758 (hartree)

Electronic energy + thermal enthalpy correction = -518.56664 (hartree)

Electronic energy + thermal free energy correction = -518.61505 (hartree)

N	-2.94598	2.20489	0.23617
H	-2.61413	2.67576	1.05358
C	-2.45599	0.81895	0.23617
C	-3.36298	-0.24116	0.23507
C	-1.08481	0.56383	0.23559
C	-2.89846	-1.55637	0.23409
H	-4.444	-0.0398	0.23507
C	-0.62006	-0.75193	0.23512
H	-0.36986	1.39937	0.23582
C	-1.52689	-1.81174	0.23451
H	-3.61316	-2.39203	0.2334
H	0.46111	-0.95284	0.23473
H	-1.16093	-2.84883	0.23415
C	-2.45378	2.89862	-0.96274
C	-3.10352	2.72455	-2.18503
C	-1.33856	3.7319	-0.87804
C	-2.63739	3.3834	-3.32266

H	-3.98303	2.06789	-2.2515
C	-0.8719	4.39064	-2.01625
H	-0.82675	3.86964	0.08548
C	-1.52126	4.21619	-3.23831
H	-3.14927	3.24618	-4.28613
H	0.00746	5.04755	-1.94924
H	-1.15371	4.73521	-4.13553

■ tert-butyl carbazole (neutral)

Electronic energy = -832.1613 (hartree)

Electronic energy + zero-point energy = -831.76169 (hartree)

Electronic energy + thermal energy correction = -831.74148 (hartree)

Electronic energy + thermal enthalpy correction = -831.74053 (hartree)

Electronic energy + thermal free energy correction = -831.80955 (hartree)

C	-3.82025	-2.59106	-0.00017
C	-2.42855	-2.59106	-0.00017
C	-1.70819	-1.38715	-0.00017
C	-2.40808	-0.19575	-0.00015
C	-3.83709	-0.19485	-0.00021
C	-4.53996	-1.3881	-0.00023
H	-1.88156	-3.54546	-0.0002
H	-0.60939	-1.39919	-0.00006
H	-5.63894	-1.39565	-0.00037
C	-3.14762	2.04418	0.00004
C	-3.29467	3.4181	0.00011
C	-4.59025	3.95641	-0.00001
C	-5.70837	3.12779	0.00022
C	-5.57038	1.73277	0.00003
C	-4.29518	1.19262	-0.00008
H	-2.41904	4.08202	0.00019
H	-4.71905	5.04888	-0.00018
H	-6.45786	1.08454	0.00002
N	-1.89159	1.21681	0.
H	-1.13571	0.7688	0.4774
C	-7.11901	3.74559	0.00049
C	-7.8806	3.28567	1.25749
H	-8.70688	3.94218	1.43404

H	-8.24178	2.28918	1.11102
H	-7.22231	3.3061	2.10078
C	-7.00675	5.2815	0.00157
H	-6.47195	5.60064	-0.86851
H	-7.98685	5.71075	-0.00478
H	-6.48345	5.60025	0.87875
C	-7.88032	3.28742	-1.25732
H	-8.76808	3.87395	-1.37033
H	-7.25694	3.41454	-2.11763
H	-8.14492	2.25542	-1.15794
C	-4.58573	-3.92733	-0.00003
C	-3.58105	-5.09446	-0.00309
H	-4.11291	-6.02291	-0.00428
H	-2.9669	-5.03411	-0.8772
H	-2.96527	-5.03729	0.8701
C	-5.46843	-4.01314	1.25897
H	-4.91898	-3.65597	2.10481
H	-6.3435	-3.41267	1.12265
H	-5.7561	-5.03041	1.42422
C	-5.47321	-4.01067	-1.25583
H	-4.90851	-3.70698	-2.11244
H	-5.80926	-5.01778	-1.38889
H	-6.31849	-3.36516	-1.13869

■ carbazole (neutral)

Electronic energy = -517.592 (hartree)

Electronic energy + zero-point energy = -517.41615 (hartree)

Electronic energy + thermal energy correction = -517.40722 (hartree)

Electronic energy + thermal enthalpy correction = -517.40627 (hartree)

Electronic energy + thermal free energy correction = -517.45011 (hartree)

C	-3.82025	-2.59106	-0.00017
C	-2.42855	-2.59106	-0.00017
C	-1.70819	-1.38715	-0.00017
C	-2.40808	-0.19575	-0.00015
C	-3.83709	-0.19485	-0.00021
C	-4.53996	-1.3881	-0.00023

H	-4.36704	-3.54558	-0.00007
H	-1.88156	-3.54546	-0.0002
H	-0.60939	-1.39919	-0.00006
H	-5.63894	-1.39565	-0.00037
C	-3.14762	2.04418	0.00004
C	-3.29467	3.4181	0.00011
C	-4.59025	3.95641	-0.00001
C	-5.70837	3.12779	0.00022
C	-5.57038	1.73277	0.00003
C	-4.29518	1.19262	-0.00008
H	-2.41904	4.08202	0.00019
H	-4.71905	5.04888	-0.00018
H	-6.71601	3.56909	0.00041
H	-6.45786	1.08454	0.00002
N	-1.89159	1.21681	0.
H	-1.13571	0.7688	0.4774

■ 4,4'-dicyanodiphenyl amine (neutral)

Electronic energy = -703.32791 (hartree)

Electronic energy + zero-point energy = -703.13274 (hartree)

Electronic energy + thermal energy correction = -703.119 (hartree)

Electronic energy + thermal enthalpy correction = -703.11806 (hartree)

Electronic energy + thermal free energy correction = -703.17463 (hartree)

N	-2.94598	2.20489	0.23617
H	-2.61413	2.67576	1.05358
C	-2.45599	0.81895	0.23617
C	-3.36298	-0.24116	0.23507
C	-1.08481	0.56383	0.23559
C	-2.89846	-1.55637	0.23409
H	-4.444	-0.0398	0.23507
C	-0.62006	-0.75193	0.23512
H	-0.36986	1.39937	0.23582
C	-1.52689	-1.81174	0.23451
H	-3.61316	-2.39203	0.2334
H	0.46111	-0.95284	0.23473
C	-2.45378	2.89862	-0.96274

C	-3.10352	2.72455	-2.18503
C	-1.33856	3.7319	-0.87804
C	-2.63739	3.3834	-3.32266
H	-3.98303	2.06789	-2.2515
C	-0.8719	4.39064	-2.01625
H	-0.82675	3.86964	0.08548
C	-1.52126	4.21619	-3.23831
H	-3.14927	3.24618	-4.28613
H	0.00746	5.04755	-1.94924
C	-1.01443	-3.26398	0.234
C	-1.00658	4.94297	-4.49469
N	-0.63288	-4.34523	0.23362
N	-0.62338	5.4841	-5.43012

■ 4DMDP-IPN (neutral)

Electronic energy = -3403.4982 (hartree)

Electronic energy + zero-point energy = -3402.4357 (hartree)

Electronic energy + thermal energy correction = -3402.3636 (hartree)

Electronic energy + thermal enthalpy correction = -3402.3627 (hartree)

Electronic energy + thermal free energy correction = -3402.554 (hartree)

C	0.08432	-1.19481	0.27193
C	1.50122	-1.17436	0.26893
C	2.22608	0.00036	0.00023
C	1.50087	1.17488	-0.2685
C	0.08399	1.19487	-0.27167
C	-0.63977	-0.00007	0.00011
C	2.23452	-2.35736	0.58663
C	2.2339	2.3581	-0.586
N	2.84953	3.29928	-0.85275
N	2.85035	-3.29833	0.85364
N	-0.58852	2.40328	-0.58136
C	-0.32549	3.58444	0.17595
C	-1.24518	2.49678	-1.85267
C	-0.35223	4.85337	-0.40887
C	-0.03238	3.4979	1.54626
C	-0.61742	2.03025	-3.01598
C	-2.499	3.09764	-1.96876
C	-0.11068	6.00388	0.34371
H	-0.55648	4.96099	-1.46654

C	0.23056	4.63559	2.29446
H	-0.00849	2.53294	2.03601
C	-1.23877	2.13886	-4.25233
H	0.36701	1.57924	-2.95974
C	-3.12397	3.22848	-3.20765
H	-2.993	3.47927	-1.08416
C	0.18782	5.90349	1.70402
H	-0.14533	6.96462	-0.15415
H	0.45814	4.55353	3.35194
C	-2.49853	2.7391	-4.35996
H	-0.75094	1.77147	-5.1486
H	-4.09581	3.70261	-3.25585
O	0.44601	6.96532	2.52847
O	-3.02716	2.80638	-5.61587
C	0.42468	8.27655	1.96868
C	-4.31213	3.40519	-5.78479
H	1.17923	8.38552	1.18427
H	0.65522	8.95433	2.788
H	-0.56221	8.51929	1.56389
H	-5.07876	2.85987	-5.22748
H	-4.5284	3.34697	-6.84929
H	-4.30434	4.45305	-5.4723
N	-2.05731	-0.00031	-0.00001
C	-2.77328	0.81841	0.92607
C	-2.77293	-0.81904	-0.92633
C	-3.96638	1.44941	0.56188
C	-2.31757	0.98726	2.24072
C	-2.31666	-0.9882	-2.24073
C	-3.96623	-1.44992	-0.56252
C	-4.68821	2.22123	1.472
H	-4.35099	1.33551	-0.44361
C	-3.01846	1.76923	3.14814
H	-1.41299	0.49043	2.56669
C	-3.01724	-1.77028	-3.14831
H	-1.4119	-0.49153	-2.56643
C	-4.68773	-2.22183	-1.47281
H	-4.35123	-1.33586	0.44281
C	-4.21329	2.39286	2.77537
H	-5.60869	2.68724	1.14398

H	-2.65307	1.89268	4.16205
C	-4.21228	-2.39374	-2.77596
H	-2.6514	-1.89395	-4.16203
H	-5.60839	-2.68772	-1.1451
O	-4.83844	3.13285	3.74173
O	-4.8371	-3.13383	-3.74244
C	-6.05733	3.79327	3.4072
C	-6.05611	-3.79421	-3.40826
H	-5.90753	4.51934	2.60291
H	-6.37129	4.31397	4.30949
H	-6.83005	3.07631	3.11478
H	-5.90661	-4.52012	-2.60376
H	-6.36969	-4.31511	-4.31056
H	-6.82896	-3.0772	-3.1163
N	-0.58783	-2.40342	0.58147
C	-1.24498	-2.49707	1.85255
C	-0.32426	-3.58457	-0.17567
C	-2.49892	-3.09776	1.96806
C	-0.61748	-2.03098	3.01617
C	-0.03059	-3.49809	-1.54586
C	-0.35111	-4.85347	0.40923
C	-3.1243	-3.22884	3.20673
H	-2.9927	-3.47905	1.0832
C	-1.23922	-2.13982	4.2523
H	0.36705	-1.58011	2.96032
C	0.23283	-4.63581	-2.29386
H	-0.00663	-2.53317	-2.03569
C	-0.10907	-6.004	-0.34315
H	-0.55583	-4.96103	1.46682
C	-2.49913	-2.73988	4.35935
H	-4.09624	-3.70282	3.2545
H	-0.75162	-1.77278	5.14883
C	0.19002	-5.90367	-1.70334
H	0.46084	-4.55378	-3.35125
H	-0.14382	-6.96471	0.15475
O	-3.02814	-2.80742	5.61508
O	0.44872	-6.96552	-2.5276
C	-4.31324	-3.40611	5.78347
C	0.4276	-8.27669	-1.96766

H	-5.07963	-2.86054	5.22608
H	-4.52981	-3.34814	6.84792
H	-4.30549	-4.45389	5.4707
H	1.18184	-8.3853	-1.1829
H	0.65872	-8.95449	-2.78679
H	-0.55939	-8.51972	-1.56327
N	3.6424	0.0006	0.00024
C	4.33744	-0.20507	1.22961
C	4.3373	0.20626	-1.22925
C	5.51244	-0.95865	1.28064
C	3.84674	0.34638	2.42228
C	3.84719	-0.34633	-2.42162
C	5.51154	0.96099	-1.28063
C	6.19405	-1.15051	2.48146
H	5.90654	-1.40549	0.37549
C	4.50615	0.13748	3.62611
H	2.94269	0.94502	2.41324
C	4.50644	-0.13743	-3.62555
H	2.94373	-0.94587	-2.41229
C	6.19301	1.15287	-2.48152
H	5.90512	1.40871	-0.37569
C	5.68999	-0.60716	3.66755
H	7.10264	-1.73889	2.47605
H	4.11948	0.56484	4.54501
C	5.68954	0.60836	-3.66733
H	4.12023	-0.56569	-4.54423
H	7.10102	1.74216	-2.47641
O	6.27423	-0.74487	4.89577
O	6.27367	0.74601	-4.8956
C	7.47982	-1.50141	4.99401
C	7.4786	1.50358	-4.99419
H	7.32373	-2.53811	4.68266
H	7.76032	-1.47828	6.04491
H	8.27847	-1.05418	4.39526
H	7.32155	2.54031	-4.68342
H	7.75917	1.48012	-6.04506
H	8.27761	1.05741	-4.39514

■ 4DP-IPN (neutral)

Electronic energy = -2487.0562 (hartree)

Electronic energy + zero-point energy = -2486.2521 (hartree)

Electronic energy + thermal energy correction = -2486.2016 (hartree)

Electronic energy + thermal enthalpy correction = -2486.2007 (hartree)

Electronic energy + thermal free energy correction = -2486.3414 (hartree)

C	-1.66504	-3.30614	-0.00108
C	-0.26988	-3.30614	-0.00108
C	0.42765	-2.09839	0.00126
C	-0.27	-0.88988	0.0024
C	-1.66483	-0.88996	0.00192
C	-2.36243	-2.09816	0.00058
C	0.5005	0.44351	0.00491
C	0.49967	-4.64007	-0.00183
N	1.07417	1.43628	0.00677
N	1.07264	-5.63325	-0.00238
N	1.89765	-2.09828	0.00211
C	2.49394	-2.19983	-1.25886
C	2.49248	-1.99665	1.26376
C	3.77926	-2.72831	-1.45619
C	1.75289	-1.80331	-2.38475
C	1.75017	-2.39327	2.3888
C	3.77749	-1.46801	1.46257
C	4.30288	-2.83452	-2.74113
H	4.35535	-3.07967	-0.60937
C	2.2801	-1.92972	-3.66389
H	0.75924	-1.38518	-2.24653
C	2.27589	-2.26679	3.66854
H	0.75674	-2.81152	2.24943
C	4.29961	-1.36173	2.74811
H	4.35452	-1.11657	0.61642
C	3.56411	-2.4381	-3.85389
H	5.29842	-3.24793	-2.8707
H	1.68539	-1.618	-4.51692
C	3.55962	-1.75825	3.86002
H	1.68024	-2.57859	4.52088
H	5.29495	-0.9482	2.87883
H	3.97952	-2.52735	-4.85179

H	3.97386	-1.66894	4.8584
N	-2.39998	-4.57923	-0.00295
C	-2.79773	-5.03544	-1.26374
C	-2.59775	-5.15458	1.25632
C	-3.89988	-5.88283	-1.45723
C	-2.09421	-4.58353	-2.39283
C	-2.55964	-4.32184	2.3873
C	-2.78067	-6.53321	1.44655
C	-4.26555	-6.2736	-2.74179
H	-4.48439	-6.21238	-0.60741
C	-2.4791	-4.96735	-3.67137
H	-1.234	-3.9331	-2.25769
C	-2.70117	-4.8499	3.6645
H	-2.42638	-3.25136	2.25469
C	-2.93784	-7.0481	2.72979
H	-2.77268	-7.20231	0.59527
C	-3.56314	-5.82372	-3.85776
H	-5.12253	-6.92809	-2.86834
H	-1.91968	-4.60181	-4.5269
C	-2.90092	-6.21731	3.84764
H	-2.66546	-4.18453	4.52154
H	-3.07622	-8.1178	2.85381
H	-3.85735	-6.13139	-4.85531
H	-3.0215	-6.62814	4.84413
N	-3.83243	-2.09792	0.00034
C	-4.42775	-1.95169	-1.25669
C	-4.42821	-2.24393	1.25717
C	-5.7128	-1.41624	-1.43616
C	-3.68595	-2.30836	-2.39535
C	-3.68666	-1.88753	2.39607
C	-5.7135	-2.77894	1.43622
C	-6.23543	-1.26453	-2.71692
H	-6.28944	-1.09489	-0.57787
C	-4.21218	-2.13667	-3.66961
H	-2.6925	-2.73137	-2.27127
C	-4.21336	-2.05904	3.67016
H	-2.69302	-1.46487	2.27231
C	-6.23661	-2.93047	2.71682
H	-6.28998	-3.10009	0.57775

C	-5.49593	-1.62156	-3.84246
H	-7.23078	-0.84654	-2.83253
H	-3.61691	-2.41819	-4.53268
C	-5.49735	-2.5737	3.84259
H	-3.61827	-1.77773	4.53343
H	-7.23214	-3.34811	2.8321
H	-5.91057	-1.49697	-4.83689
H	-5.91235	-2.69814	4.83689
N	-2.40015	0.38291	0.00312
C	-2.56462	0.97687	-1.2523
C	-2.83149	0.82035	1.2595
C	-2.74304	2.35815	-1.42686
C	-2.49608	0.161	-2.39409
C	-2.15804	0.35177	2.40009
C	-3.93878	1.66471	1.43616
C	-2.86626	2.89202	-2.70605
H	-2.758	3.01456	-0.56584
C	-2.60384	0.70794	-3.66663
H	-2.36591	-0.91133	-2.27386
C	-2.57697	0.71656	3.67349
H	-1.29424	-0.29653	2.27825
C	-4.33865	2.03636	2.7162
H	-4.50063	2.0068	0.57608
C	-2.79926	2.0779	-3.83472
H	-3.00179	3.96344	-2.81784
H	-2.54505	0.05536	-4.53219
C	-3.66596	1.57002	3.84366
H	-2.04033	0.33841	4.53807
H	-5.199	2.68885	2.8296
H	-2.89348	2.50347	-4.82787
H	-3.98674	1.86284	4.83749

■ 4tcz-IPN (neutral)

Electronic energy = -3740.6216 (hartree)

Electronic energy + zero-point energy = -3739.007 (hartree)

Electronic energy + thermal energy correction = -3738.9159 (hartree)

Electronic energy + thermal enthalpy correction = -3738.915 (hartree)

Electronic energy + thermal free energy correction = -3739.1407 (hartree)

C	-1.20629	1.34595	-0.00234
C	-1.20605	-0.05741	-0.00233
C	0.00001	-0.765	0.
C	1.20608	-0.05737	0.00234
C	1.20627	1.34596	0.00234
C	-0.00002	2.0372	-0.00001
C	-2.44575	-0.77266	-0.00474
C	2.44576	-0.77264	0.00474
N	3.44738	-1.3476	0.00668
N	-3.44738	-1.34759	-0.00669
N	0.00005	-2.235	0.
C	-0.00003	-3.0353	-1.13181
C	-0.00002	-3.03539	1.13175
C	0.	-2.6925	-2.47946
C	-0.00016	-4.39322	-0.7259
C	-0.00016	-4.39327	0.72574
C	0.00002	-2.6927	2.47944
C	-0.0001	-3.72126	-3.41841
H	0.00008	-1.65546	-2.80135
C	-0.00025	-5.39802	-1.69317
C	-0.00024	-5.39816	1.69292
C	-0.00007	-3.72153	3.41829
H	0.00011	-1.65569	2.80141
C	-0.00022	-5.08267	-3.05629
H	-0.00011	-3.44399	-4.4649
H	-0.00033	-6.43545	-1.3742
C	-0.00021	-5.08292	3.05606
H	-0.00033	-6.43556	1.37385
H	-0.00006	-3.44437	4.46481
C	-0.00009	-6.21579	-4.10122
C	-0.0001	-6.21608	4.10095
C	-1.2598	-7.09213	-3.91703
C	1.26044	-7.09103	-3.91756
C	-0.00071	-5.68448	-5.5457
C	-1.25989	-7.09231	3.91689
C	-0.00055	-5.68475	5.54542
C	1.26033	-7.09143	3.91724
H	-1.27644	-7.90333	-4.65209
H	-1.29978	-7.54655	-2.92433

H	-2.17064	-6.50104	-4.04594
H	1.27739	-7.90238	-4.65244
H	2.17072	-6.4992	-4.04708
H	1.3013	-7.54513	-2.92475
H	-0.88641	-5.08018	-5.76038
H	0.88451	-5.07967	-5.761
H	-0.00068	-6.52258	-6.24801
H	-1.3	-7.54671	2.92419
H	-1.27654	-7.9035	4.65194
H	-2.17067	-6.50113	4.04587
H	-0.00052	-6.52283	6.24774
H	0.88472	-5.07998	5.76062
H	-0.88621	-5.08038	5.76016
H	2.17068	-6.49969	4.04671
H	1.27724	-7.90278	4.65212
H	1.30111	-7.54554	2.92442
N	2.48162	2.07698	0.00482
C	3.17857	2.4743	-1.12562
C	3.17337	2.47571	1.13794
C	2.88431	2.303	-2.47386
C	4.35572	3.14985	-0.71736
C	4.35238	3.15075	0.73426
C	2.87293	2.3061	2.48503
C	3.77902	2.81406	-3.41102
H	1.98537	1.78706	-2.79753
C	5.22971	3.64898	-1.6829
C	5.22193	3.65109	1.70318
C	3.76332	2.81833	3.42566
H	1.9725	1.79057	2.80522
C	4.9593	3.49133	-3.04656
H	3.54093	2.67551	-4.45799
H	6.129	4.16511	-1.36215
C	4.94527	3.49513	3.06577
H	6.12269	4.16681	1.3859
H	3.52043	2.68111	4.4717
C	5.94468	4.05442	-4.08953
C	5.92581	4.05949	4.11261
C	7.33106	3.39759	-3.90172
C	6.07656	5.58341	-3.90649

C	5.48742	3.78875	-5.53493
C	7.313	3.4023	3.93217
C	5.46173	3.79568	5.55616
C	6.0587	5.58823	3.92828
H	8.04478	3.7862	-4.63535
H	7.74284	3.58953	-2.90818
H	7.27162	2.31334	-4.03012
H	6.7737	6.00124	-4.64
H	5.11066	6.07864	-4.03855
H	6.44786	5.84532	-2.91297
H	5.40422	2.71967	-5.74916
H	4.52294	4.25573	-5.75276
H	6.21611	4.20521	-6.23579
H	7.72947	3.59294	2.94032
H	8.0233	3.7918	4.66865
H	7.25286	2.31822	4.06167
H	6.18713	4.21299	6.25993
H	4.49625	4.26299	5.76883
H	5.37745	2.72688	5.77135
H	5.09225	6.08372	4.05521
H	6.75244	6.0069	4.66454
H	6.43468	5.84888	2.93619
N	-0.00002	3.5072	-0.00001
C	0.00271	4.30751	-1.13181
C	-0.00288	4.30757	1.13174
C	0.00606	3.96472	-2.47946
C	0.0016	5.66542	-0.72589
C	-0.00197	5.66546	0.72574
C	-0.00617	3.96487	2.47942
C	0.00829	4.99349	-3.41839
H	0.00695	2.92769	-2.80136
C	0.0039	6.67024	-1.69314
C	-0.00445	6.67034	1.69294
C	-0.00858	4.99369	3.41828
H	-0.00688	2.92786	2.80139
C	0.00729	6.3549	-3.05626
H	0.01092	4.71624	-4.46488
H	0.00302	7.70767	-1.37417
C	-0.00778	6.35509	3.05607

H	-0.00375	7.70774	1.37388
H	-0.01112	4.71652	4.4648
C	0.00953	7.48804	-4.10117
C	-0.01066	7.48823	4.10097
C	1.26863	8.36459	-3.91388
C	-1.2516	8.36305	-3.92061
C	0.01379	6.95674	-5.54566
C	1.24943	8.36468	3.92001
C	-0.01367	6.95689	5.54543
C	-1.27079	8.36337	3.91416
H	1.28694	9.1758	-4.64888
H	1.30608	8.819	-2.92108
H	2.17988	7.77366	-4.04055
H	-1.26689	9.17441	-4.65553
H	-2.16145	7.77106	-4.05238
H	-1.29499	8.81713	-2.9279
H	0.90013	6.35259	-5.75817
H	-0.87078	6.35178	-5.76315
H	0.01535	7.79484	-6.24795
H	1.2919	8.81911	2.92742
H	1.26412	9.17587	4.65512
H	2.15999	7.77367	4.05124
H	-0.01558	7.79496	6.24776
H	-0.89937	6.35196	5.75844
H	0.87155	6.35267	5.76234
H	-2.18135	7.77146	4.04139
H	-1.28965	9.1747	4.64901
H	-1.3092	8.81748	2.92125
N	-2.48167	2.0769	-0.00481
C	-3.17362	2.47528	-1.13794
C	-3.1785	2.47442	1.12562
C	-2.87334	2.30536	-2.48502
C	-4.35264	3.15032	-0.73426
C	-4.35576	3.14977	0.71737
C	-2.88404	2.3035	2.47387
C	-3.76392	2.81726	-3.42566
H	-1.9729	1.78986	-2.80521
C	-5.22238	3.65031	-1.70317
C	-5.22968	3.64904	1.6829

C	-3.77867	2.81469	3.41103
H	-1.98499	1.78775	2.79755
C	-4.94588	3.49404	-3.06577
H	-3.52113	2.67984	-4.47169
H	-6.12316	4.16601	-1.38592
C	-4.95906	3.49176	3.04656
H	-6.12907	4.16498	1.36213
H	-3.54041	2.67647	4.458
C	-5.9269	4.05767	-4.11256
C	-5.94454	4.05459	4.08958
C	-6.06141	5.58627	-3.92806
C	-7.31337	3.39899	-3.93211
C	-5.46254	3.79463	-5.55617
C	-6.07812	5.58336	3.90583
C	-5.48635	3.79027	5.53492
C	-7.33028	3.39621	3.90267
H	-6.75543	6.00428	-4.66443
H	-6.43792	5.84648	-2.93606
H	-5.09545	6.08277	-4.05473
H	-8.02422	3.78793	-4.66835
H	-7.25212	2.315	-4.06192
H	-7.7298	3.5889	-2.9401
H	-4.49742	4.26278	-5.76863
H	-5.37738	2.72596	-5.77171
H	-6.18825	4.21154	-6.25985
H	-6.45035	5.84445	2.91245
H	-6.77525	6.0008	4.63958
H	-5.11267	6.07966	4.03706
H	-6.21503	4.20661	6.23587
H	-5.40218	2.72141	5.74988
H	-4.52212	4.25818	5.75189
H	-7.26968	2.3121	4.03185
H	-8.04422	3.78461	4.6362
H	-7.74246	3.58697	2.90906

■ **4Cz-IPN (neutral)**

Electronic energy = -2482.3385 (hartree)

Electronic energy + zero-point energy = -2481.619 (hartree)

Electronic energy + thermal energy correction = -2481.5732 (hartree)

Electronic energy + thermal enthalpy correction = -2481.5723 (hartree)

Electronic energy + thermal free energy correction = -2481.7003 (hartree)

C	-4.65528	0.47758	-0.18291
C	-4.65504	-0.92577	-0.18054
C	-3.44898	-1.63336	-0.18052
C	-2.24291	-0.92573	-0.18287
C	-2.24271	0.4776	-0.18523
C	-3.44901	1.16883	-0.18524
C	-5.89474	-1.64101	-0.17815
C	-1.00322	-1.641	-0.18286
N	-0.0016	-2.21596	-0.18287
N	-6.89638	-2.21595	-0.17622
N	-5.93066	1.20854	-0.18291
C	-6.62381	1.60327	-1.31552
C	-6.62162	1.60708	0.94971
C	-6.31003	1.42042	-2.66229
C	-7.80247	2.2797	-0.90745
C	-7.80108	2.28214	0.54164
C	-6.30524	1.42876	2.29649
C	-7.19588	1.92602	-3.60725
H	-5.40623	0.90175	-2.96697
C	-8.6747	2.77742	-1.88028
C	-8.67142	2.78313	1.51448
C	-7.18927	1.93753	3.24145
H	-5.40085	0.91111	2.60115
C	-8.36701	2.59808	-3.22349
H	-6.97582	1.79754	-4.66224
H	-9.58236	3.29827	-1.5914

C	-8.36114	2.6083	2.85769
H	-9.57964	3.30301	1.2256
H	-6.96718	1.8126	4.29643
H	-9.03718	2.98047	-3.98618
H	-9.02985	2.99326	3.62038
N	-3.44894	-3.10336	-0.17804
C	-3.45001	-3.90292	-1.30932
C	-3.44782	-3.89911	0.95591
C	-3.45132	-3.54202	-2.6567
C	-3.44958	-5.2612	-0.89896
C	-3.44818	-5.25877	0.55013
C	-3.44653	-3.53368	2.30208
C	-3.4522	-4.56359	-3.59994
H	-3.45164	-2.50048	-2.96312
C	-3.45049	-6.26708	-1.87011
C	-3.44722	-6.26137	1.52466
C	-3.4456	-4.55207	3.24875
H	-3.44627	-2.49111	2.605
C	-3.4518	-5.91321	-3.21391
H	-3.45323	-4.31055	-4.65536
H	-3.45018	-7.31308	-1.57946
C	-3.44593	-5.90298	2.86727
H	-3.44747	-7.30834	1.23754
H	-3.44458	-4.29548	4.30331
H	-3.45251	-6.68608	-3.97531
H	-3.44517	-6.67328	3.63126
N	-3.44901	2.63883	-0.18772
C	-3.44792	3.43839	0.94356
C	-3.4501	3.43458	-1.32167
C	-3.44662	3.0775	2.29094
C	-3.44831	4.79668	0.5332
C	-3.44971	4.79424	-0.91589

C	-3.4514	3.06915	-2.66784
C	-3.44571	4.09907	3.23418
H	-3.44633	2.03596	2.59737
C	-3.44737	5.80256	1.50434
C	-3.45064	5.79684	-1.89042
C	-3.45231	4.08754	-3.61451
H	-3.4517	2.02658	-2.97075
C	-3.44608	5.44869	2.84815
H	-3.44469	3.84604	4.2896
H	-3.44765	6.84855	1.2137
C	-3.45194	5.43844	-3.23303
H	-3.45036	6.84381	-1.6033
H	-3.45333	3.83095	-4.66907
H	-3.44534	6.22156	3.60954
H	-3.45267	6.20875	-3.99702
N	-0.96737	1.20861	-0.18769
C	-0.27425	1.60719	0.94359
C	-0.27642	1.60338	-1.32164
C	-0.58802	1.42886	2.29098
C	0.90439	2.28231	0.53326
C	0.90299	2.27987	-0.91584
C	-0.59279	1.42051	-2.6678
C	0.2978	1.93767	3.23423
H	-1.4918	0.91117	2.59739
C	1.77658	2.78334	1.50441
C	1.77332	2.77763	-1.89035
C	0.29122	1.92615	-3.61447
H	-1.49715	0.9018	-2.97073
C	1.4689	2.6085	2.84822
H	0.07774	1.81273	4.28964
H	2.68422	3.30326	1.21378
C	1.46305	2.59827	-3.23296

H	2.68151	3.29852	-1.60322
H	0.06913	1.79766	-4.66902
H	2.13905	2.99349	3.60962
H	2.13174	2.98069	-3.99694

■ 4DCDP-IPN (neutral)

Electronic energy = -3225.2457 (hartree)

Electronic energy + zero-point energy = -3224.4516 (hartree)

Electronic energy + thermal energy correction = -3224.3866 (hartree)

Electronic energy + thermal enthalpy correction = -3224.3857 (hartree)

Electronic energy + thermal free energy correction = -3224.5615 (hartree)

C	-1.20629	1.34595	-0.00234
C	-1.20605	-0.05741	-0.00233
C	0.00001	-0.765	0.
C	1.20608	-0.05737	0.00234
C	1.20627	1.34596	0.00234
C	-0.00002	2.0372	-0.00001
C	-2.44575	-0.77266	-0.00474
C	2.44576	-0.77264	0.00474
N	3.44738	-1.3476	0.00668
N	-3.44738	-1.34759	-0.00669
N	0.00005	-2.235	0.
C	-0.08837	-2.82769	-1.25891
C	0.08848	-2.82767	1.25893
C	-0.60549	-4.11822	-1.46169
C	0.30772	-2.07695	-2.38018
C	-0.30762	-2.07691	2.38019
C	0.60562	-4.11819	1.46173
C	-0.70086	-4.64189	-2.74123
H	-0.96455	-4.69932	-0.62237
C	0.20109	-2.59696	-3.65772
H	0.71385	-1.07971	-2.23963
C	-0.20099	-2.5969	3.65774
H	-0.71377	-1.07969	2.23962
C	0.701	-4.64183	2.74128

H	0.9647	-4.69929	0.62242
C	-0.29696	-3.89384	-3.85674
H	-1.10826	-5.63616	-2.88405
H	0.51384	-2.00539	-4.51033
C	0.29709	-3.89377	3.85677
H	-0.51374	-2.00532	4.51033
H	1.10842	-5.63609	2.88412
C	-0.398	-4.43848	-5.17229
C	0.39814	-4.43838	5.17233
N	-0.47963	-4.87858	-6.23792
N	0.47977	-4.87846	6.23797
N	2.48162	2.07698	0.00482
C	2.93755	2.46912	1.26273
C	3.05409	2.27431	-1.25109
C	3.79778	3.5628	1.45734
C	2.47039	1.77064	2.39039
C	2.21858	2.22611	-2.38129
C	4.43313	2.46418	-1.44136
C	4.19013	3.92698	2.73562
H	4.13295	4.14944	0.61203
C	2.854	2.14273	3.66643
H	1.80873	0.92011	2.25626
C	2.73726	2.37121	-3.65558
H	1.14988	2.08481	-2.25052
C	4.9494	2.62082	-2.71787
H	5.10626	2.45549	-0.59409
C	3.72925	3.22297	3.85768
H	4.84856	4.77717	2.87198
H	2.48655	1.59128	4.52409
C	4.11228	2.58082	-3.84246
H	2.07822	2.33428	-4.51523
H	6.01619	2.75951	-2.85088
C	4.13657	3.60309	5.17188
C	4.64998	2.74238	-5.15482
N	4.46569	3.91027	6.23642
N	5.08449	2.8729	-6.21787
N	-0.00002	3.5072	-0.00001

C	-0.14373	4.09988	1.25381
C	0.14369	4.09987	-1.25383
C	-0.66929	5.3904	1.43364
C	0.20265	3.34913	2.39141
C	-0.20269	3.34912	-2.39142
C	0.66924	5.3904	-1.43367
C	-0.82085	5.91406	2.70776
H	-0.99109	5.9715	0.57935
C	0.03993	3.86914	3.66303
H	0.61459	2.3519	2.26885
C	-0.03997	3.86912	-3.66304
H	-0.61462	2.35189	-2.26886
C	0.8208	5.91405	-2.70779
H	0.99105	5.9715	-0.57938
C	-0.46641	5.16601	3.83995
H	-1.23416	6.90833	2.83252
H	0.31487	3.27756	4.52856
C	0.46636	5.16599	-3.83997
H	-0.31491	3.27754	-4.52857
H	1.2341	6.90832	-2.83256
C	-0.62523	5.71064	5.14978
C	0.62518	5.71061	-5.14981
N	-0.75365	6.15073	6.21079
N	0.7536	6.15071	-6.21082
N	-2.48167	2.0769	-0.00481
C	-3.07574	2.23651	1.24642
C	-2.91602	2.50671	-1.25804
C	-4.45792	2.42077	1.41854
C	-2.25978	2.15425	2.38887
C	-2.42927	1.84235	-2.39796
C	-3.77318	3.60587	-1.4345
C	-4.99614	2.53907	2.69011
H	-5.11637	2.43763	0.55993
C	-2.80037	2.26108	3.65783
H	-1.18894	2.01678	2.27234
C	-2.791	2.25266	-3.66867
H	-1.7697	0.98808	-2.27807

C	-4.14361	4.00834	-2.70782
H	-4.12307	4.16691	-0.57783
C	-4.17849	2.4652	3.82722
H	-6.06511	2.67387	2.80884
H	-2.15621	2.19826	4.5272
C	-3.66324	3.33828	-3.84242
H	-2.40864	1.72716	-4.53605
H	-4.79992	4.86234	-2.82989
C	-4.73875	2.58734	5.13439
C	-4.04801	3.75776	-5.15142
N	-5.19154	2.68594	6.19325
N	-4.35888	4.09683	-6.21176

Supplementary References

1. Singh, V. K. et al. Highly efficient organic photocatalysts discovered via a computer-aided-design strategy for visible-light-driven atom transfer radical polymerization. *Nat. Catal.* **1**, 794-804 (2018).

2. Kwon, Y. et al. Formation and degradation of strongly reducing cyanoarene-based radical anions towards efficient radical anion-mediated photoredox catalysis. *Nat. Commun.* **14**, 92 (2023).

3. Back, J. et al. Visible light curable acrylic resins toward UV-light blocking adhesives for foldable displays. *Adv. Mater.* **35**, 2204776 (2022).

4. Roth, H. G., Romero, N. A. & Nicewicz, D. A. Experimental and calculated electrochemical potentials of common organic molecules for applications to single-electron redox chemistry. *Synlett* **27**, 714–723 (2016).

5. Sahari, A., Puumi, J., Mannisto, J. K. & Repo, T. Dual nickel photocatalysis for o-aryl carbamate synthesis from carbon dioxide. *J. Org. Chem.* **88**, 3822–3829 (2023).

6. Kretzschmar, A., Patze, C., Schwaebel, S. T. & Bunz, U. H. F. Development of thermally activated delayed fluorescence materials with shortened emissive lifetimes. *J. Org. Chem.* **80**, 9126–9131 (2015).

7. Etherington, M. K. et al. Persistent dimer emission in thermally activated delayed fluorescence materials. *J. Phys. Chem. C* **123**, 11109–11117 (2019).

8. Dadashi-Silab, S., Atilla Tasdelen, M., Mohamed Asiri, A., Bahadar Khan, S. & Yagci, Y. Photoinduced atom transfer radical polymerization using semiconductor nanoparticles. *Macromol. Rapid Commun.* **35**, 454–459 (2014).

9. Dadashi-Silab, S. et al. Photochemically mediated atom transfer radical polymerization using polymeric semiconductor mesoporous graphitic carbon nitride. *Macromol. Chem. Phys.* **215**, 675–681 (2014).

10. Kütahya, C., Schmitz, C., Strehmel, V., Yagci, Y. & Strehmel, B. Near-infrared sensitized photoinduced atom-transfer radical polymerization (ATRP) with a copper(II) catalyst concentration in the ppm range. *Angew. Chem. Int. Ed.* **57**, 7898–7902 (2018).

11. Kütahya, C. et al. Carbon dots as a promising green photocatalyst for free radical and ATRP-based radical photopolymerization with blue LEDs. *Angew. Chem. Int. Ed.* **59**, 3166–3171 (2020).

12. Zhang, W. et al. Atom transfer radical polymerization driven by near-infrared light with recyclable upconversion nanoparticles. *Macromolecules* **53**, 4678–4684 (2020).

13. Lu, Z. et al. A covalent organic framework as a photocatalyst for atom transfer radical polymerization under white light irradiation. *Polym. Chem.* **12**, 183–188 (2021).

14. Dadashi-Silab, S. et al. Conjugated cross-linked phenothiazines as green or red light heterogeneous photocatalysts for copper-catalyzed atom transfer radical polymerization. *J. Am. Chem. Soc.* **143**, 9630–9638 (2021).

15. Sun, M. et al. Assemblies of polyacrylonitrile-derived photoactive polymers as blue and green light photo-cocatalysts for Cu-catalyzed ATRP in water and organic solvents. *Front. Chem.* **9**, 1–11 (2021).

16. Dadashi-Silab, S. et al. Red-light-induced, copper-catalyzed atom transfer radical polymerization. *ACS Macro Lett.* **11**, 376–381 (2022).

17. Qiao, L. et al. Ultrafast visible-light-induced ATRP in aqueous media with carbon quantum dots as the catalyst and its application for 3D printing. *J. Am. Chem. Soc.* **144**, 9817–9826 (2022).

18. Luo, X. et al. A porphyrin-based organic network comprising sustainable carbon dots for photopolymerization. *Angew. Chem.*

Int. Ed. **61,** e202208180 (2022).

19. Szczepaniak, G. et al. Open-air green-light-driven ATRP enabled by dual photoredox/copper catalysis. *Chem. Sci.* **13**, 11540–11550 (2022).

20. Kapil, K. et al. Visible-light-mediated controlled radical branching polymerization in water. *Angew. Chem. Int. Ed.* **62**, e202217658 (2023).

21. Kapil, K. et al. Fully oxygen-tolerant visible-light-induced ATRP of acrylates in water: toward synthesis of protein-polymer hybrids. *Macromolecules* **56**, 2017–2026 (2023).

22. Fang, W. W. et al. Conjugated cross-linked phosphine as broadband light or sunlight-driven photocatalyst for large-scale atom transfer radical polymerization. *Nat. Commun.* **14**, 2891 (2023).

23. Ti, Q. et al. Hybrid of organophotoredox- and Cu-mediated pathways enables atom transfer radical polymerization with extremely low catalyst loading. *Macromolecules* ***56***, 2017-2026 (2023).

24. Hu, X. et al. Red-light-driven atom transfer radical polymerization for high-throughput polymer synthesis in open air. *J. Am. Chem. Soc.* **145**, 24315–24327 (2023).

25. Gierschner, J., Cornil, J. & Egelhaaf, H. J. Optical bandgaps of π-conjugated organic materials at the polymer limit: Experiment and theory. *Adv. Mater.* **19**, 173–191 (2007).

26. Gierschner, J. et al. Luminescence in crystalline organic materials: from molecules to molecular solids. *Adv. Opt. Mater.* **9**, 2002251 (2021).

27. Shi, J. et al. Solid state luminescence enhancement in π-conjugated materials: unraveling the mechanism beyond the framework of AIE/AIEE. *J. Phys. Chem. C* **121**, 23166–23183 (2017).

28. Ishimatsu, R. et al. Solvent effect on thermally activated delayed fluorescence by 1,2,3,5-tetrakis(carbazol-9-yl)-4,6-dicyanobenzene. *J. Phys. Chem. A* **117**, 5607–5612 (2013).

29. Romero, N. A. & Nicewicz, D. A. Organic photoredox catalysis. *Chem. Rev.* **116**, 10075–10166 (2016).

30. Würth, C., Grabolle, M., Pauli, J., Spieles, M. & Resch-Genger, U. Comparison of methods and achievable uncertainties for the relative and absolute measurement of photoluminescence quantum yields. *Anal. Chem.* **83**, 3431–3439 (2011).

31. Furukawa, T., Nakanotani, H., Inoue, M. & Adachi, C. Dual enhancement of electroluminescence efficiency and operational stability by rapid upconversion of triplet excitons in OLEDs. *Sci. Rep.* **5**, 8429 (2015).

32. Vadrucci, R., Weder, C. & Simon, Y. C. Organogels for low-power light upconversion. *Mater. Horizons* **2**, 120–124 (2015).
